# Influence of the Computer-Aided Decision Support System Design on Ultrasound-Based Breast Cancer Classification

**DOI:** 10.3390/cancers14020277

**Published:** 2022-01-06

**Authors:** Zuzanna Anna Magnuska, Benjamin Theek, Milita Darguzyte, Moritz Palmowski, Elmar Stickeler, Volkmar Schulz, Fabian Kießling

**Affiliations:** 1 Institute for Experimental Molecular Imaging, Uniklinik RWTH Aachen and Helmholtz Institute for Biomedical Engineering, Faculty of Medicine, RWTH Aachen University, 52074 Aachen, Germany; zmagnuska@ukaachen.de (Z.A.M.); benjamin.theek@gmail.com (B.T.); mdarguzyte@ukaachen.de (M.D.); vschulz@ukaachen.de (V.S.); 2 Radiologie Baden-Baden, Beethovenstraße 2, 76530 Baden-Baden, Germany; moritz.palmowski@radiologie-baden-baden.de; 3 Department of Obstetrics and Gynecology, University Clinic Aachen, RWTH Aachen University, Pauwelsstr. 30, 52074 Aachen, Germany; estickeler@ukaachen.de; 4 Comprehensive Diagnostic Center Aachen, Uniklinik RWTH Aachen, Pauwelsstr. 30, 52074 Aachen, Germany; 5 Physics Institute III B, RWTH Aachen University, 52074 Aachen, Germany; 6 Hyperion Hybrid Imaging Systems GmbH, 52074 Aachen, Germany; 7 Fraunhofer Institute for Digital Medicine MEVIS, Am Fallturm 1, 28359 Bremen, Germany

**Keywords:** breast cancer, ultrasound, medical image analysis, machine learning, deep learning, radiomics

## Abstract

**Simple Summary:**

The implementation of artificial intelligence in the computer-aided decision (CAD) support systems holds great promise for future cancer diagnosis. It is crucial to build these algorithms in a structured manner to ensure reproducibility and reliability. In this context, we used a dataset of breast ultrasound (US) images with 252 breast cancer and 253 benign cases to refine the CAD image analysis workflow. Various dataset preparations (i.e., pre-processing, and spatial augmentation) and machine learning algorithms were tested to establish the framework with the best performance in the detection and classification of breast lesions in US images. The efficacy of the proposed workflows was evaluated regarding accuracy, precision, specificity, and sensitivity.

**Abstract:**

Automation of medical data analysis is an important topic in modern cancer diagnostics, aiming at robust and reproducible workflows. Therefore, we used a dataset of breast US images (252 malignant and 253 benign cases) to realize and compare different strategies for CAD support in lesion detection and classification. Eight different datasets (including pre-processed and spatially augmented images) were prepared, and machine learning algorithms (i.e., Viola–Jones; YOLOv3) were trained for lesion detection. The radiomics signature (RS) was derived from detection boxes and compared with RS derived from manually obtained segments. Finally, the classification model was established and evaluated concerning accuracy, sensitivity, specificity, and area under the Receiver Operating Characteristic curve. After training on a dataset including logarithmic derivatives of US images, we found that YOLOv3 obtains better results in breast lesion detection (IoU: 0.544 ± 0.081; LE: 0.171 ± 0.009) than the Viola–Jones framework (IoU: 0.399 ± 0.054; LE: 0.096 ± 0.016). Interestingly, our findings show that the classification model trained with RS derived from detection boxes and the model based on the RS derived from a gold standard manual segmentation are comparable (*p*-value = 0.071). Thus, deriving radiomics signatures from the detection box is a promising technique for building a breast lesion classification model, and may reduce the need for the lesion segmentation step in the future design of CAD systems.

## 1. Introduction

Breast cancer is the leading cause of death among women (30% of all cancers in females) [1]. Imaging performed with X-ray, MRI, and US is the basis of its detection. Although X-ray mammography is used as a primary screening tool, US is usually performed as a follow-up to gather more diagnostic information. MRI is only used for special cases (e.g., high-risk genetic mutation, multifocal disease), and its value for screening is currently under debate due to its high costs and the need for contrast agents [2]. One of the biggest challenges of US imaging is its high operator dependence [3]. This problem not only considers the repeatability of measurement, but also the user expertise. 

Recently, the idea of “computers helping doctors” has increasingly become a reality due to the evolution of artificial intelligence (AI) technologies. The implementation of AI in CAD systems holds great promise for the future of cancer detection [4]. These systems are designed to support first-line tumor diagnosis by providing a second, potentially objective, opinion on the content of medical images.

The training process of machine or deep learning algorithms, embedded in CAD, is strongly influenced by the quality and quantity of the dataset [5,6]. Hence, the first step in the CAD system is dataset preparation. Pre-processing is an operation that suppresses undesired noise or enhances image features [7]. For medical images, this significantly increases the ability to interpret their content, even for non-imaging experts [8]. Image pre-processing also considers geometric transformations, which are widely used in data augmentation—a technique devoted to enlarge and diversify the images dataset [9]. Because AI-based CAD systems need big and diverse datasets for training that are difficult to obtain, data pre-processing can overcome this issue. For instance, using spatial transformations with other augmentation techniques noticeably improved skin cancer detection [10]. Furthermore, Zhang et al. proposed a very interesting and successful image augmentation approach by extending the dataset of breast US images with the BIRADS-oriented feature maps [11]. Including these maps in the training of breast lesion classification frameworks can improve the accuracy of a breast cancer diagnosis. Nevertheless, it is often not enough to use data pre-processing for enlarging a dataset for a particular task. Here, transfer learning is another technique that can help to overcome the data shortage in the development of AI-based CAD systems. In this method, the network is trained with the use of a dataset that is not necessarily composed of medical images [12,13]. Thus, the algorithm is exposed to a broader spectrum of information that improves its generalization capabilities. The training results in obtaining a robust pre-trained network, which can be further fine-tuned to develop a task-specific detection or classification algorithms.

The lesion needs to be found before it can be classified. However, many CAD frameworks concentrate solely on lesion classification rather than detection [14,15]. For example, Han et al. presented a CAD system for breast cancer classification, where lesion detection was still performed manually by clinicians [16]. The localization of the lesion with a point or detection box constitutes the base for a segmentation step in the CAD system, and without segmentation, the lesion may not be classified [17,18]. However, the segmentation of a breast lesion is still challenging because tumor margins are often not completely covered in US images and disturbed by artifacts. To better address the lesion segmentation task, authors have focused on developing new or refining already existing algorithms [19,20]. For example, Xue et al. proposed a deep convolutional neural network equipped with a global guidance block that enhances breast lesion segmentation by utilizing the broad contextual features of US images [21]. Nonetheless, from the clinical perspective, detailed lesion segmentation, however, is not required to diagnose breast cancer. Indeed, clinicians localize and measure the size of lesions to monitor growth, to perform tumor staging, and to control the therapy outcome, but for decisions about tumor type, the analysis of the border zone and the adjacent tissue is of pivotal importance [22]. Thus, detection and area definition should be carefully incorporated in CAD.

To diagnose breast cancer, the doctor analyzes a few parameters related to lesion size, shape, and echo pattern. In the early days of CAD systems, the embedded machine learning algorithms were trained to search only for the same features that the clinician would look for [15,23]. However, images are more than pictures, and they can be used to extract multiple powerful features that are not visible to the naked eye [24,25]. This became possible with the introduction of radiomics analysis, which tries to exploit the full information content of medical images for cancer diagnosis [26,27,28]. For this purpose, radiomics analysis can provide new parameters reflecting important characteristics of the tumor microenvironment. For instance, using textural features extracted from US images, differentiation between triple-negative breast cancer, invasive ductal carcinoma, and fibroadenoma can be improved [29,30]. However, mining the image-based features is a complicated task that involves multiple image processing steps (i.e., segmentation, feature extraction), which have a potential influence on the developed tumor classification model [31]. Introducing deep learning into CAD frameworks enabled the automated derivation of descriptive features [32]. Therefore, omitting the feature extraction and selection steps makes these systems more user independent. Although deep learning algorithms often represent a black box, and the ability to explain results remains a critical issue, they already outperform the machine learning-based CAD systems in the sensitivity of cancer diagnosis [33]. Furthermore, it has been shown that the sensitivity of deep learning CAD systems increases when using both handcrafted and automatically derived features [34].

**Figure 1:**
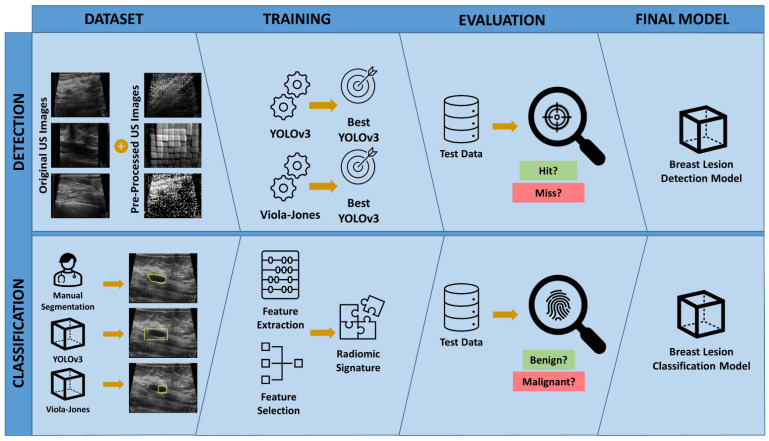
Study overview. To develop the breast lesion detection model, various datasets combining the original and pre-processed US images were prepared and used to train and select the best YOLOv3 and Viola–Jones classifiers. The final model was selected by evaluating the performance of trained detection models on the test dataset. To develop the breast lesion classification model, the breast lesions were outlined in US images by an expert radiologist (Manual Segmentation), the best YOLOv3 breast lesion detection model, and the best Viola–Jones breast lesion detection model. Three separate RS were obtained using the features extracted from the manually delineated and automatically outlined breast lesion segments. The best breast lesion classification model was selected by evaluating the performance of each RS on the test dataset.

In this study (Figure 1), we investigated the advantage of image pre-processing as a data augmentation technique and assessed the influence of the training dataset composition on the performance of deep learning and machine learning-based breast lesion detection algorithms. We hypothesized that an effective radiomics signature for breast cancer classification can be extracted from lesion detection bounding boxes alone by omitting the segmentation task.

## 2. Materials and Methods

### 2.1. Internal and Public Dataset

The retrospective study was approved by the Institutional Review Board (or Ethics Committee) of the University Hospital of the RWTH Aachen International University (EK 066/18) and was conducted according to the guidelines of the Declaration of Helsinki. 

The study collective includes ultrasound images of 119 female patients who were identified in the database of the Department of Obstetrics and Gynecology, University Clinic Aachen and the “Radiologie Baden-Baden” diagnosis center. In 71 patients, 77 breast cancer lesions were detected and documented by US. All the breast cancers were confirmed histopathologically. In 48 patients, the diagnosis of 50 benign lesions was made with US. Diagnosis of benign lesions was confirmed in 12 patients by histology (5 fibroadenomas and 7 cysts) and in 36 patients by follow up studies. In the latter cases, the follow up included at least one follow up carried out after 12 months.

The used US images were acquired with an Acuson Antares ultrasound system (Siemens-Healthineers, Erlangen, Germany) equipped with a 13.5 MHz transducer (VFX13-5). Results were stored in DICOM format (Reitz-CS computer systems, Dresden, Germany). For the study, images were retrospectively reviewed by a breast radiologist with 11 years of experience (M.P.) with knowledge about the histologic results and/or the follow-up studies, and anonymized for the following analysis.

To extend the assembled study collective, breast US images from two publicly available datasets were used [17,35]. The first dataset was collected at the UIDAT Diagnostic Center of the Parc Taulí Corporation, Sabadell Spain. It comprises 163 US images generated from different female patients in which 110 benign and 53 malignant lesions were found. The second dataset was obtained and provided by the Department of Radiology of Thammasat University and Queen Sirkit Center of Breast Cancer of Thailand. The study collective includes US images of 249 female patients with the diagnosis of 62 benign solid mass lesions, 21 fibroadenomas, 22 cysts, and 144 cancer lesions. The dataset provides the manual segmentations of the documented lesions drawn by 3 clinicians from the Department of Radiology of Thammasat University.

### 2.2. Dataset Preparation and Sampling

The patient data, identified in the database of the Department of Obstetrics and Gynecology, University Clinic Aachen and the “Radiologie Baden-Baden” diagnosis center, were exported from the DICOM format. All samples were saved as 8-bit grayscale images, normalized, and cropped to the size of 600 × 700 pixels. Discrete wavelet transform was used for speckle noise removal [36]. The images from the public dataset were reviewed. The examples comprising caliper measurements embedded in the image were excluded. The final dataset was composed of 497 patients/505 lesions (Table 1).

The prepared dataset was divided into 2 data pools. The first data pool (234 patients/235 lesions) was used for developing the breast lesion detection functions. The second data pool (263 patients/270 lesions) was used for developing the breast lesion classification model.

### 2.3. Dataset Augmentation

The images from the first data pool were augmented spatially and by computing their exponential, logarithm, Laplacian of Gaussian, square root, squared, and wavelet derivatives. All the augmentation scenarios are listed and described in Table 2 and captured in Figures S1–S6. The final number of augmented images derived from one original image was 118 (109 spatial and 9 filtered/processed). Data augmentation was performed in MATLAB (Version 2020a, The Math Works Inc. MATLAB, Natick, MA, USA). The derived augmentation scenarios were used for building 8 training datasets (Table 3).

### 2.4. Breast Lesion Detection

The patients from the first data pool were divided into training, validation, and test groups using random sampling (Table 4). The ground truth was labeled based on the US images with caliper measurements taken by the expert radiologist (M.P.) in the training dataset. For the validation and test dataset, 3 users (radiologist, physician, and ultrasound expert) were asked to detect the lesions by marking them with a bounding box. The labelling was performed using MATLAB software.

The breast lesion detection functions were developed using Viola–Jones and YOLOv3 algorithms. The first, Viola–Jones, computes the feature descriptor with a sliding window, and this results in object detection [37]. The second, YOLOv3, is a convolutional neural network that solves a single regression problem to localize objects [38]. Both algorithms follow the underlying gray-level patterns of the images to localize the objects of interest. The Viola–Jones and YOLOv3 algorithms were trained with 8 assembled datasets (Table 3).

The Viola–Jones classifiers were trained from scratch. In every image, 1 positive and 4 negative regions of interests were marked (Figure S7). The negative regions of interests were cropped from the original image and included in the pool of negative samples. All classifiers were trained using histograms of oriented gradients features [39]. The size of the object being searched for was set automatically by the Viola–Jones algorithm. The inference was performed with 10, 15, 20, and 25 stages. The experiment was implemented with MATLAB software.

The YOLOv3 classifiers were trained using the open-source Python library ImageAI [40]. This library provides classes and methods for training new detection models on any type of image without a need for any additional adjustments on the used dataset. The ImageAI library is built on the Tensorflow backbone. The pre-trained YOLOv3 network (i.e., base model), provided by the ImageAI developers, was trained with the COCO dataset [13]. The custom detection functions were trained with two different transfer learning strategies. First, all detection layers of the pre-trained YOLOv3 network were frozen and the new models were trained on top of it. Second, the new detection functions were obtained with the so-called “fine tuning” of the base model by retraining the pre-trained YOLOv3 network on the new dataset with a very low learning rate (i.e., 0.001). During this process, the pre-trained features incrementally adapt to the new data. Only the positive examples had to be provided for the training. The positive annotations were issued in Pascal VOC format. The size of the objects being searched for was set automatically by the algorithm. The inference was performed with stochastic gradient descent with the learning rate of 0.01 (transfer learning by “freezing layers”) and 0.001 (“fine tuning”), and batch size of 4. Each model was trained with 10, 15, 20, and 25 epochs. The experiment was implemented in the Python programming language.

### 2.5. Evaluation and Performance Metrics for Breast Lesion Detection

The intersection over union (IoU) and localization error (LE) were used to evaluate the accuracy of breast lesion detection functions. Both Viola–Jones and YOLOv3 algorithms compute the coordinates of the found detection boxes. Therefore, they were used to calculate IoU and LE.

IoU is the gold standard metric to evaluate object detection models [13,41]. The “overlap criterion” states that the detection bounding box, which has IoU greater than 0.5 with the ground truth bounding box, is a true positive finding. Otherwise, it is considered a false positive. A case where no lesion is detected is considered a false negative [18]. No criteria for the true negative detections were established. The IoU was calculated with the following formula:

(1) $$\mathrm{IoU}\;=\frac{Area\;of\;Overlap}{Area\;of\;Union}$$

LE measures the disagreement in the localization and size of the detection box and ground truth box. Thus, we hypothesized that it could be used as a supporting evaluation metric together with IoU. The detection is classified as true positive when its LE, with reference to ground truth, is less than 0.1. This means that the detection box is localized less than 10 pixels from the ground truth box, and its width and height are less than 10% smaller/bigger than the original. Detections that do not meet the criteria are considered false positives. A case where no lesion is detected is considered a false negative. No criteria for true negative detections were established. The LE was calculated with the following formula:

(2) $$\mathrm{LE}={\left(xc_{gt}-xc_{d}\right)}^{2}+{\left(yc_{gt}-yc_{d}\right)}^{2}+{\left(w_{gt}-w_{d}\right)}^{2}+{\left(h_{gt}-h_{d}\right)}^{2}$$

where *xc_gt_*, *yc_gt_* are coordinates of the center of the ground truth bounding box; *xc_d_*, *yc_d_* are coordinates of the center of the detection bounding box; *w_gt_*, *h_gt_* are width and height of the ground truth bounding box; *w_d_*, *h_d_* are width and height of the detection bounding box.

The final values of IoU and LE per image were computed as the mean of IoU and LE scores calculated separately for all the users. The final IoU and LE of the lesion detection algorithm were calculated as the mean of all the IoU and LE scores obtained for every image in the test dataset. Additionally, the standard deviation was calculated. Together with IoU and LE, recall (3), precision (4), and *F*1-score (5) were computed. Furthermore, the robustness of the detection algorithms was assessed with the recall-IoU and recall-LE curves [42].

(3) $$recall=\frac{\left|TP\right|}{\left|TP\right|+\left|FN\right|}$$

(4) $$precision=\frac{\left|TP\right|}{\left|TP\right|+\left|FP\right|}$$

(5) $$F1=\frac{2\cdot \left(Recall\cdot Precision\right)}{Recall+Precision}$$

When no false negative (*FN*) samples were obtained in the detection process, the recall (6) was calculated as a quotient of the total number of true positive (*TP*) findings and the total number of ground truths in the dataset (*N*):

(6) $$recall=\frac{\left|TP\right|}{N}$$

### 2.6. Detecting Breast Lesions in the Classification Dataset

The second data pool was used to develop the breast lesion classification models. The patients comprising the data pool were divided into two subsets: Feature Selection and Classification. In each group the patients were randomly sampled. Thus, the benign and malignant examples were equally distributed. In the Classification Subset, the patients were sampled into training and test groups (Table 5). The images comprising the second data pool were not augmented.

**Figure 2:**
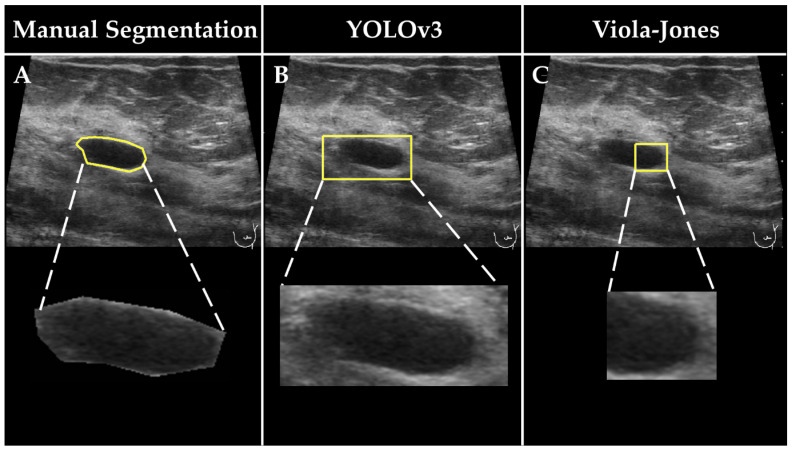
Examples of detected lesions in US images obtained by (**A**) expert radiologist, (**B**) and the YOLOv3 and (**C**) Viola–Jones detection models. The bottom row shows the magnifications of corresponding areas that were considered for further classification.

The developed detection functions (i.e., the best Viola–Jones and the best YOLOv3 models) were applied to localize breast lesions in both subsets of the second data pool. The obtained detection boxes were used to solely outline (i.e., “segment”) breast lesions in the images. The ground truth segments were obtained manually by a radiologist with 20 years of experience (F.K.) in breast US imaging (Figure 2). The images and the corresponding binary representations (i.e., masks) of the segments outlined by the expert radiologist, and YOLOv3 and Viola–Jones detection functions, were assembled in 3 separate datasets named in the following manner: “Manual Segmentation”, “YOLOv3”, and “Viola–Jones”. For the samples that were not detected by the YOLOv3 or Viola–Jones models, the segments in the size of the image were computed. The Manual Segmentation, YOLOv3, and Viola–Jones datasets were later used to develop 3 independent breast lesion classification models.

### 2.7. Radiomics Signature Extraction for Breast Lesion Classification

The radiomics features were calculated with the PyRadiomics software [43], which is an open-source package for mining radiomics features from medical images. The histogram-based (with *binWidth: 25*), textural (Gray Level Co-occurrence Matrix (GLCM), Gray Level Size Zone Matrix (GLSZM), Gray Level Run Length Matrix (GLRLM), Gray Level Dependence Matrix (GLDM), Neighboring Gray Tone Difference Matrix (NGTDM), and wavelet (with *‘coif1′* wavelet) features were calculated from original and derived images (i.e., Laplacian of Gaussian, squared, square root, logarithm, exponential, gradient, and Local Binary Pattern). The shape-based features were not extracted. All the considered groups of features were previously thoroughly described [27]. Radiomics features were extracted separately for the datasets based on Manual Segmentations, YOLOv3, and Viola–Jones. Hence, 3 separate sets of features were formed. The breast lesions outlined by radiologists, YOLOv3, and Viola–Jones were used separately to extract radiomics features. Thus, 3 separate datasets of features—Ground Truth, YOLOv3 and Viola–Jones—were obtained. In total, 1023 features per dataset were mined and the values of extracted features were normalized. The least absolute shrinkage and selection operator (LASSO) with L1 regularization was used for the feature selection task [44]. This is a supervised algorithm that identifies features that are strongly correlated with the response variable (benign or malignant). Moreover, LASSO determines the features that are loosely associated with the response. It is important to identify the most and least descriptive traits for the classification task because the latter, in particular, can promote the overfitting of the trained model. In the training process of LASSO, the magnitude of the penalty coefficient lambda is determined. LASSO utilizes this coefficient both to select the most descriptive features for the classification task, and to remove the least descriptive traits.

The optimal magnitude of the penalty coefficient, lambda, was determined with the 10-fold-cross-validation search performed on the Feature Selection Subset of Manual Segmentation, YOLOv3, and Viola–Jones datasets. Finally, LASSO was used to find 3 separate effective RS for Manual Segmentation, YOLOv3, and Viola–Jones datasets.

### 2.8. Evaluation and Performance Metrics for Breast Lesion Classification

To identify the best algorithm for the breast lesion classification, the Classification Lerner App, a MATLAB built-in application was used. The Manual Segmentation, YOLOv3, and Viola–Jones RS, selected from the training groups of Classification Subsets of the second data pool, were trained with the 5- and 10-fold-cross-validation and 20%, 25%, and 30% holdout. All the trainings were done once per set of conditions. After each training, we selected the 3 best performing breast lesion classification functions per RS, which were later applied to the test groups of Classification Subsets. The selected breast lesion classification models were evaluated by calculating and comparing their sensitivity (7), specificity (8), and accuracy (9). Additionally, the Receiver Operating Characteristic curves (ROC) were drawn, and the area under the Receiver Operating Characteristic curve (AUROC) was computed. Finally, the best breast lesion classification model was chosen concerning the calculated evaluation metrics.

(7) $$Sensitivity=\frac{\left|TP\right|}{\left|TP\right|+\left|TN\right|}\;$$

(8) $$Specificity=\frac{\left|TN\right|}{\left|TN\right|+\left|FP\right|}$$

(9) $$Accuracy=\frac{\left|TP\right|+\left|TN\right|}{\left|TP\right|+\left|FP\right|+\left|TN\right|+\left|FN\right|}$$

Here, the correct classification was considered as true positive (*TP*). The incorrectly classified sample was assigned as false positive (*FP*). The data accurately assigned as negative was counted as true negative (*TN*), and as false negative (*FN*) in the opposite case. Finally, we conducted DeLong’s test to statistically evaluate the performance of models trained with YOLOv3- and Viola–Jones-derived RS by comparing their AUCROC to the Manual Segmentation RS-based model, which in this case is considered a gold standard [45].

## 3. Results

### 3.1. IoU and LE Utilized for Lesion Detection Evaluation

**Figure 3:**
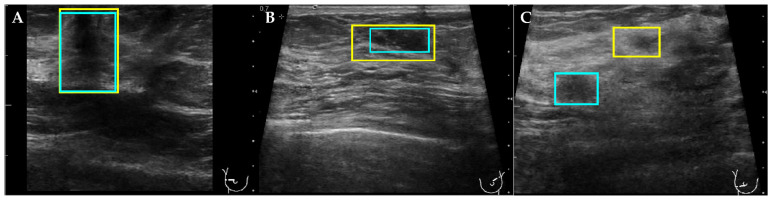
Evaluation of the breast lesion detection using IoU and LE scores. The automatically obtained detection boxes (in yellow) and ground truth boxes (in blue) were used to calculate the IoU and LE scores for 3 different evaluation scenarios. First, (**A**) where both IoU and LE satisfy the threshold conditions (IoU = 0.88 and LE = 0.0004); second, (**B**) where only LE does and IoU is below the threshold (IoU = 0.48 and LE = 0.01); and third, (**C**) where IoU is equal to 0 and LE still satisfy the threshold conditions (IoU = 0 and LE = 0.07).

We hypothesized that LE can be used additionally to IoU as a detection evaluation metric. The joint use of LE and IoU can be demonstrated in three different scoring situations (Figure 3). First, if both values satisfy their threshold conditions (i.e., IoU > 0.5 and LE < 0.1), the detection is marked as true positive. Second, if IoU is slightly lower than the threshold (i.e., indicating the false positive detection) and LE still indicates the true positive area, the detection is marked as true positive. Third, if the IoU value is close to 0, and LE still indicates the true positive detection, then the detection is classified as false positive. 

### 3.2. YOLOv3 Trained with Logarithmic Images Obtained Best Breast Lesion Detection

**Figure 4:**
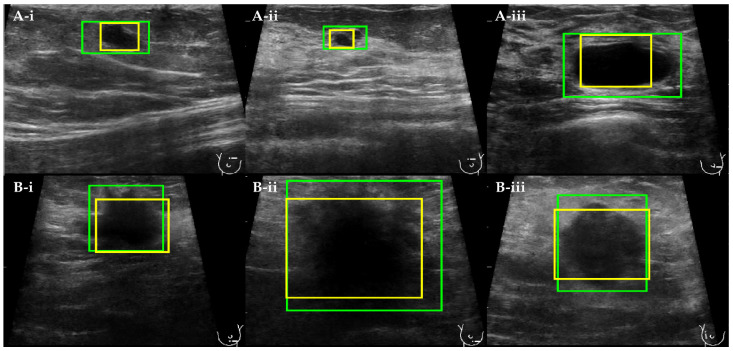
The comparison between automatically computed detections of (**A**(**i**–**iii**)) benign and (**B**(**i**–**iii**)) malignant breast lesions obtained with YOLOv3 (green) and Viola–Jones (yellow) detection models.

The detection boxes obtained by YOLOv3 are visually bigger than those found by the Viola–Jones algorithm (Figure 4). Furthermore, they comprise not only the breast lesion but also the neighboring tissue. The detection bounding boxes computed by the Viola–Jones algorithm are tighter and, in some cases, bound only part of the breast lesion.

In the validation process, the eight best Viola–Jones and the eight best YOLOv3 breast lesion detection functions were revealed. Each of these was trained on one of the eight assembled datasets (Table 3). YOLOv3 detection models obtained with transfer learning by “fine tuning” and “freezing layers” were evaluated on the validation dataset. It was revealed that “fine tuning” produces classifiers with higher final IoU and lower LE in comparison to the “freezing layers” method. Moreover, these detection functions obtained higher precision, recall, and F1-score (Tables S1 and S2).

The best Viola–Jones and the best YOLOv3 detection functions were trained on the dataset including spatially augmented images and exponential or logarithmic derivatives of original US images, respectively. Both obtained the highest final IoU and the lowest LE (Table 6 and Table 7), and they were more powerful with respect to precision, recall and F1-score in comparison to other classifiers (Tables S3 and S4).

The eight best detection functions trained with Viola–Jones and the eight best trained with YOLOv3 were applied to the test group. The classifier that obtained the highest final IoU and the lowest LE for the YOLOv3-based detection was trained with the dataset including spatially augmented and logarithmic derivatives of original images. For the Viola–Jones based detection, the best results were obtained with the dataset including all the provided augmentations of original US images (Table 8 and Table 9). The final IoU calculated for Viola–Jones has a rather low value. By comparison, the final LE score for the same detection function is close to zero. This indicates that the algorithm produces detection bounding boxes that are smaller than the ground truth; however, the obtained detections nonetheless correctly localize the breast lesion. The settings chosen for Viola–Jones obtained high recall, but low precision and F1-score, when calculated with reference to the IoU score (Table S5). This shows that, when scored only with IoU, the detection function produces a large number of false positives. However, using LE as a supporting evaluation metric increases the values of precision and the F1-score, and hence reduces the number of false positives. The best Viola–Jones-based detection function obtained low values of all the evaluation metrics calculated with reference to IoU (Table S6). In this case, the change in precision score values is more outstanding for Viola–Jones than for the best YOLOv3 detection function. Thus, here, it is shown that YOLOv3 produces detection bounding boxes that are more similar to the ground truth, both in size and location.

The robustness of both YOLOv3 and Viola–Jones breast lesion detection functions was evaluated by plotting the recall-IoU and recall-LE characteristics. Detection methods that propose more true positive windows in a similar location (i.e., high recall and high IoU) are more reproducible [42]. Thus, they are more likely to find a true positive object in a test dataset, similar to the one that they were validated on. In our study, a similar analysis can be conducted while comparing the recall-LE characteristics of YOLOv3- and Viola–Jones-based detection functions. The lower LE score indicates a higher probability of true positive detection, thus, the recall decreases with the value of LE going towards zero. The recall-IoU and recall-LE characteristics drawn for two training strategies of YOLOv3 show that transfer learning by “fine tuning” exhibits better reproducibility in comparison to the second method (Figure S8). Although both schemes present a gradual decrease in the recall value with increasing IoU score, the magnitude of recall for transfer learning by “freezing layers” is lower than in the case of transfer learning by “fine tuning”. For both algorithms, the recall calculated with reference to LE changes more gradually than the one calculated with reference to IoU (Figure S8). However, the Viola–Jones-based detections are still less reproducible than the ones obtained with YOLOv3.

**Figure 5:**
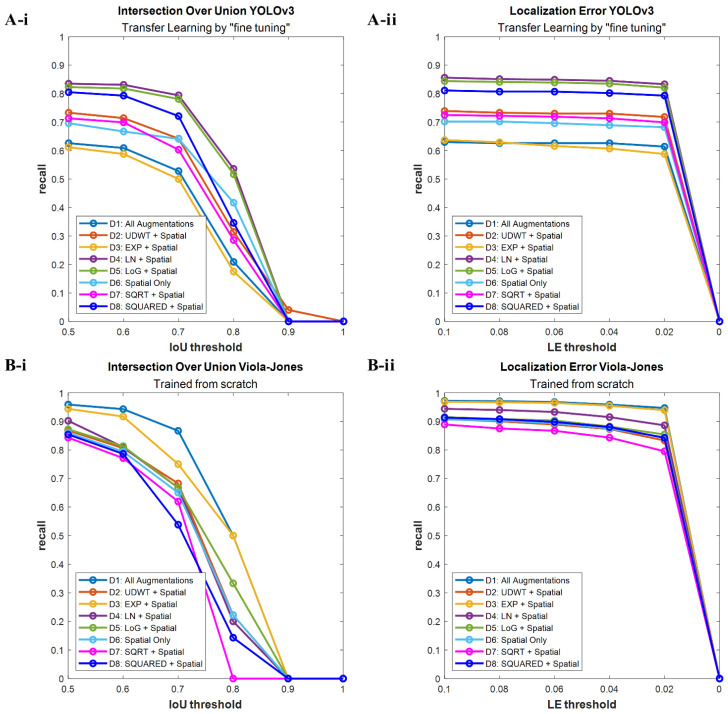
Recall-IoU and recall-LE curves resulting from the evaluation of the best breast lesion detection algorithms on the test group. (**A-i**) YOLOv3 detection functions scored with different IoU thresholds; (**A-ii**) YOLOv3 detection functions scored with different LE thresholds; (**B-i**) Viola–Jones detection functions scored with different IoU thresholds; (**B-ii**) Viola–Jones detection functions scored with different LE thresholds.

The IoU-recall and LE-recall characteristics plotted for both algorithms, with respect to the detection results obtained on the test dataset, imply that YOLOv3 is likely the more reproducible and robust algorithm for breast lesion detection (Figure 5). In order to verify our hypothesis, the eight best detection functions trained with Viola–Jones and YOLOv3 were applied to the second data pool. Thus, the advantage of using YOLOv3 for breast lesion detection over the Viola–Jones algorithm could be confirmed. The performance of the detection models was compared by plotting the recall-IoU and recall-LE characteristics (Figure S9). In addition, the evaluation metrics (recall, precision, and F1-score) with respect to IoU and LE scores were calculated (Tables S7 and S8). The performed experiment revealed that YOLOv3, trained with the dataset including spatially augmented and logarithmic derivatives of original images, is the best breast lesion detection strategy. The model obtained the highest final IoU and the lowest LE scores. The best results for the Viola–Jones-based detection were obtained with the same dataset. The new settings chosen for the Viola–Jones-based detection obtained higher recall than the YOLOv3 model but, at the same time, a very low precision score, which implies that the chosen Viola–Jones classifier computes a high number of false positive detections in comparison to the YOLOv3 breast lesion detection algorithm. Furthermore, the optimal training settings for the developed YOLOv3 function were the same at each step of the evaluation (i.e., validation, test, and test on the second data pool), whereas, for the Viola–Jones localization algorithm, the optimal training scenario was always changing. Therefore, with this experiment, we confirmed the higher robustness and reproducibility of the selected YOLOv3-based breast lesion detection.

### 3.3. Selected Radiomics Signatures for Brest Lesion Classification

The three RS selected separately for the Manual Segmentation, YOLOv3, and Viola–Jones datasets comprised 33, 51, and 41 descriptive features, respectively (Table 10). The different number of radiomics features selected for each Classification Subset was caused by the following factors: First, all the extracted “segments” have different characteristics. Those outlined by YOLOv3 are bigger and comprise not only the lesion itself, but also the surrounding tissue. By comparison, the segments produced by Viola–Jones are smaller and mainly embed the inner part of the lesion. The segments obtained by the expert radiologist represent the complete shape but miss the tissue adjacent to the tumor. Second, in the feature selection process, different values of the penalty coefficient lambda were found for each of the utilized classification datasets. All of the above resulted in a varying number of features in the obtained RS. Most of the selected features were computed from the derivatives of original US images rather than from the original images themselves (Tables S9–S11).

### 3.4. Breast Lesion Classification Using Image-Based Features

The three best-performing breast lesion classification models, with the highest accuracy, sensitivity, specificity and AUROC for every training scenario, were chosen in the validation step and evaluated on classification test groups (Table S12). We tested several classification algorithms because the applied breast lesion detection methods outlined tissue areas with different RS. In this context, we found that each RS obtains the best classification outcome when combined with a different classification method. In particular, the best model for the Manual Segmentation dataset was trained with 5-, 10-fold-cross-validation, or with 30% holdout validation, and the Weighted KNN classifier. This model provided the best sensitivity and specificity for discriminating the breast lesion type (Table 11). Thus, it is likely to classify both malignant and benign samples as true positives in a similar dataset. The function based on the Ensemble Subspace KNN model, which was trained with a 5-fold-cross-validation, resulted in the best breast lesion classification for the YOLOv3 dataset. The chosen model obtained the second-best overall accuracy, sensitivity, and specificity among all selected breast lesion classification strategies. Finally, for the Viola–Jones dataset, the Median KNN algorithm trained with 5-fold-cross-validation or 30% holdout was found to acquire the best classification skills. However, in comparison to the other models, it obtained the lowest values of the precision metrics.

**Figure 6:**
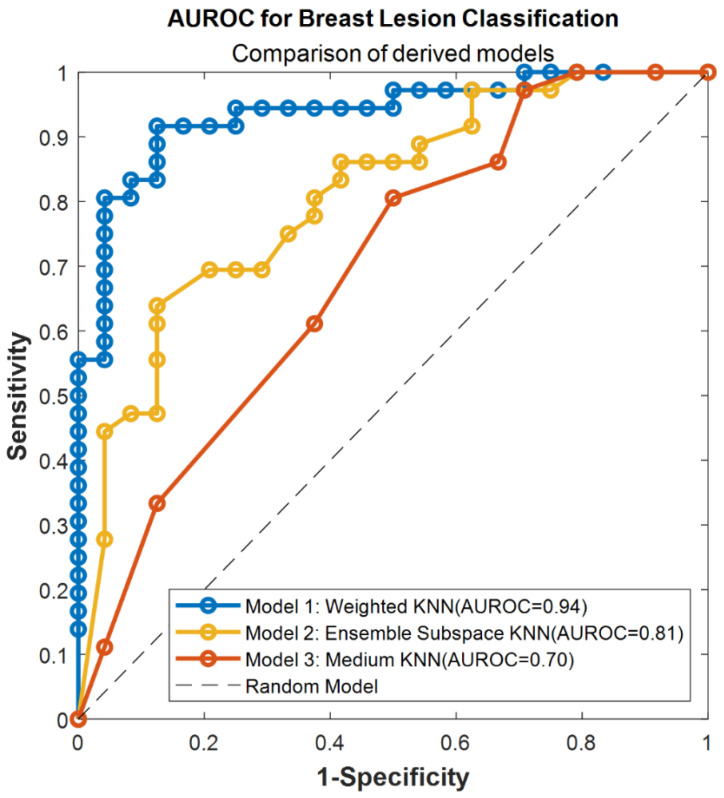
The AUROC curves of breast lesion classification models trained for Manual Segmentation (Model 1), YOLOv3 (Model 2), and Viola–Jones (Model 3) derived radiomics signatures. The dotted line presents the model with no discriminative capacity (Random Model).

All classifiers can distinguish between malignant and benign breast lesions (AUROC > 0.5) (Figure 6). The Weighted KNN classifier trained on the Manual Segmentation dataset obtained the highest AUROC (0.94). The second-best was the Ensemble Subspace KNN model based on inference with the YOLOv3 dataset (AUROC = 0.81), and the last was the Median KNN classifier trained on the Viola–Jones dataset (AUROC = 0.70). We conducted DeLong’s test to statistically compare the AUROC curves (Table 12). Our findings show that there is no statistically significant difference between the AUROC of YOLOv3 and the Manual Segmentation (*p*-value = 0.071). Furthermore, the AUROC computed for the Viola–Jones-based model is significantly different from the gold standard (*p*-value = 0.002). The obtained results show that the model derived with the YOLOv3-based dataset and the Ensemble Subspace KNN classifier may be considered, as an alternative to the gold standard Manual Segmentation-derived RS, for breast lesion classification in US images. Nevertheless, additional experiments have to be conducted for the further improvement of the developed classification method.

## 4. Discussion

In this study, we methodically analyzed the different steps a CAD system should consider to detect and classify benign or malignant breast lesions in US images. First, we found that computing pre-processed images is a valid data augmentation technique for a dataset of US images. Including these images in the training dataset improves the performance of breast lesion detection models trained with YOLOv3 and Viola–Jones algorithms. Moreover, we found that YOLOv3-based breast lesion detection is more robust and reproducible in comparison to the Viola–Jones-based detection. In the second part of our study, we discovered that the effective RS can be extracted solely from the detection of bounding boxes. The obtained model achieved promising results in the classification of both malignant and benign breast lesions.

Data augmentation prevents overfitting and can provide different information that can be extracted from the original dataset [7,8,9]. In our study, we used a broad selection of spatial augmentations to build a versatile training dataset for developing the breast lesion detection strategy. Some of the used transformations may not represent the typical presentation of US images with an transducer on top; however, these images are still highly useful to expand the learning abilities of the detection algorithms. Furthermore, we expanded the heterogeneity of the training dataset by including the pre-processed images. Application of imaging filters created a new matrix of grey levels. Hence, the algorithm faced a different pool of features that could be learned. We found that using image filtering methods for the data augmentation, along with the spatial transformations, can improve the performance of breast lesion detection. In particular, the inclusion of logarithmic images, derived from the original US data, for the training of the YOLOv3 algorithm, improves its performance in breast lesion detection. By comparison, the Viola–Jones-based model for breast lesion localization benefits from being trained on a dataset with all the presented augmentation of the original US data. Furthermore, our study showed that YOLOv3 is a better choice than Viola–Jones for developing breast lesion detection functions. YOLOv3 models express higher robustness and reproducibility of breast lesion detection in US images. Furthermore, these models obtained higher scores while being evaluated with reference to the gold standard IoU and proposed LE. IoU is one of the most popular and most reliable metrics used for the evaluation of object detection models [13,41]. However, we showed that it is not ideal for analyzing breast lesion detection in US images. The detection boxes that are smaller than or encompassed by the ground truth bounding box compute a lower score with respect to IoU, even though the lesion was detected correctly. Thus, it results in a high number of false positive detections. The LE score calculated for the same detection boxes classifies them as true positives. Often, where the IoU will discard a positive sample, the LE helps to preserve it. LE considers the seeding point plus the size of the detected bounding box, which makes it more robust than seed-point-based evaluation [17,18]. Nevertheless, using LE alone can be also misleading. One can see that neither IoU nor LE is an ideal measure for scoring the breast lesion detection. However, in combination, they can give a better overview of the detection function performance. Our findings suggest that using LE as a supporting score for IoU is beneficial for the evaluation of the breast lesion detection algorithm.

Typically, the lesion detection is followed by the segmentation task in CAD systems. Segmentation is much more complex than drawing a bounding box around a region of interest. In US imaging, in particular, one needs to analyze images obtained with different transducer positions, to capture the whole shape of the lesion. This can be challenging for any segmentation algorithm, as it cannot work with a well-arranged series of images as in CT or MRI [16,46]. Moreover, using bounding boxes may be more real-time capable. Thus, during the examination, a region of interest could be simultaneously analyzed with a changing transducer position [47,48].

Developers of classical machine learning or deep learning-based segmentation models aim to obtain a detailed outline of the tumor [16,49] because the identified segments constitute the base for the last element of CAD, which is the lesion classification [46,50]. Generating the accurate segment of a breast lesion provides an opportunity to compute the morphological shape features, which were reported to have more discriminative power over the textural traits [51]. However, these features are frequently computed with respect to 2D US images. In our opinion, it would be more reliable to use 3D US images to assess the breast tumor shape morphology. Thus far, it has been shown that there is no significant difference between extracting textural radiomics features from the whole lesion or just a part of it [52]. Furthermore, the inclusion of textural features enables the capture of characteristics of breast lesions not only at the microscale, but also the macroscale, i.e., by quantifying its gray level zones. Moreover, bounding boxes comprise the breast lesion and adjacent tissue, which is not the case for accurate segmentation. Thus, the selected features reflect the underlying characteristics of the breast lesion and its neighboring tissue. In the clinic, a doctor diagnoses the breast lesion, while simultaneously analyzing its surrounding tissue, and segmenting is not important for this task.

We investigated whether the generated detection bounding boxes, representing “segments”, can be applied for the breast lesion classification. First and foremost, it is of high importance to derive the RS that will explain well a particular classification problem. Using a large number of features can lead to overfitting; thus, it is favorable to use feature selection methods to identify the most descriptive and reproducible traits [53,54]. In our study, we reduced the obtained features space with the LASSO model. This resulted in the identification of three RS comprising traits that were most correlated with either malignant or benign breast lesions. Our results show that the classification of benign and malignant breast lesions with these RS derived from just the detection box is a promising and robust alternative. In particular, the sensitivity and specificity of the breast lesion classification model, based on the features derived from the YOLOv3 dataset, are similar to those obtained by other groups [55,56]. Our model obtained balanced values of sensitivity and specificity, which implies that it has almost equal ability in discriminating malignant and benign breast lesions. This is also the case for RS derived from the Manual Segmentation dataset. The classification model based on the gold standard manual segmentation-derived RS obtained higher values of sensitivity and specificity in comparison to the YOLOv3 model. Furthermore, its overall accuracy was higher than that of the other developed breast lesion classification functions. However, drawing the ROC curves has an advantage over calculating the overall accuracy in describing the performance of the classification model [57]. ROC graphs are plotted for different classification thresholds of machine learning or deep learning algorithms. Thus, they indicate the robustness of the developed classification function. Furthermore, ROC curves allow the calculation of AUROC, which represents the discriminative ability of a model. The value of AUROC indicates how likely it is that the classifier will rank a randomly selected true positive sample higher than the negative sample [58]. Therefore, the classification model with a higher AUROC is more likely to classify a truly positive sample correctly. In our study, the Manual Segmentation dataset-derived classification had the highest AUROC of all the developed breast lesion classification models. The second best AUROC was obtained by the classification model built with RS selected from the YOLOv3 dataset. However, the statistical analysis of AUROC curves obtained for RS derived from YOLOv3 detection bounding boxes and gold standard manual segmentations revealed that these two breast lesion classifications models are comparable. Therefore, both models can be used for the task, regardless of class distribution or misclassification costs indicated by the precision metrics. The opposite conclusion was established for the RS derived from the Viola–Jones dataset. Furthermore, this model obtained the lowest value of AUROC. The final classification outcome of RS derived from Viola–Jones detection bounding boxes may have been worsened by the high number of false positive samples comprised in the Viola–Jones classification dataset.

In the presented study, we concluded that the bounding boxes that comprise the breast lesion and adjacent tissue are promising candidates for building the breast cancer classification model. Furthermore, the classification results obtained using these bounding boxes for building effective RS are statistically comparable to those computed by RS derived from the accurate segments. In the future, it would be interesting to compare the performance of our breast lesion classification method with a deep learning classification network. This would include the evaluation of whether a combination of YOLO3-based lesion detection followed by CNN-based lesion characterization is superior to the use of areas segmented by alternative methods or to a CNN-based analysis of the entire US image. Moreover, the generalizability of the obtained RS may be increased by incorporating additional statistical [59] or filtering feature selection methods [60]. Finally, the performance of our breast lesion classification model may be improved by using unsupervised classification algorithms [61].

Finally, we would like to mention the limitations of our study. First, our dataset was small, which may limit the strength of our conclusion. Second, some of the cases were not found in the second data pool with the selected detection methods, hence resulting in the extraction of the descriptive features from the whole image instead of the localized region of interest. Improving the performance of our breast lesion detection method will be an important issue for future studies because it has a direct influence on the extracted RS. Third, using classical machine learning and handcrafted features may have influenced the developed breast lesion classification models [4,32]. Finally, our study did not investigate the classification between different benign breast lesion types. Although the utilized dataset included patients with histologically proven cysts and fibroadenomas, they were not considered as separate classes in the lesion classification task due to small sub-cohorts. Building a balanced dataset with more examples of different benign breast lesion phenotypes would expand the classification abilities of the proposed algorithm. This sub-analysis, however, will be performed once our data repository has sufficiently grown.

## 5. Conclusions

In this study, we methodically analyzed the different steps a CAD system should consider to detect and classify benign or malignant breast lesions in US images. First, we showed that utilizing image pre-processing as data augmentation, along with spatial transformations, is recommended for developing breast lesion detection functions. Second, we suggest using LE together with IoU to improve the evaluation of breast lesion detection in US images. Third, breast lesion detection with the YOLOv3 algorithm is more robust and reproducible in comparison to the Viola–Jones-based algorithm. Fourth, the effective RS for breast cancer classification can be extracted solely from detection bounding boxes, omitting the segmentation task. Therefore, in the future, this may enhance the comprehensive assessment of the breast lesion type in US images without the need to segment the lesion in CAD systems, which will save time and may pave the way for real-time analysis. Our study provides a basis for further research. This may comprise the investigation of further descriptive features, and deep learning or unsupervised classification methods, and the construction of balanced datasets to improve our preliminary results and allow the subclassification of cancers and benign lesions.

## Figures and Tables

**Table 1:** Characteristics of the datasets included in the study.

Characteristics	Study Collective	UIDAT	Rodtook
Total number of patients	119	163	215
Total number of lesions	127	163	215
Subtypes			
Malignant	77 (60.6%)	53 (32.5%)	122 (56.7%)
Benign	38 (29.9%)	71 (43.6%)	53 (24.7%)
Cyst	7 (5.5%)	0 (0.0%)	21(9.8%)
Fibroadenoma	5 (4.0%)	39 (23.9%)	19 (8.8%)

**Table 2:** Augmentation scenarios.

Name	Description	No. of Images
A1: Flips	The image was flipped about its origin, x-axis, and y-axis	3
A2: Rotation	The image was rotated clockwise and counterclockwise by 45º, 90º, and 135º. New pixels were filled symmetrically at the edges.	6
A3: Shear	Original, flipped about the origin, flipped about the x-axis and flipped about y-axis images were sheared by 5º, 10º, 15º, 20º, 25º, and 30º. New pixels were filled symmetrically at the edges.	96
A4: Translation	The image was translated right (x-axis) and down (y-axis), right (x-axis) and up (y-axis), left (x-axis) and up (y-axis) and left (x-axis) and down (y-axis) by 10% of its width and height.	4
A5: UDWT	The single decomposition with UDWT using coif2 wavelet was applied to the original image. All resultant decomposition matrices were included in the dataset.	4
A6: EXP	The exponential derivative of the original image was computed	1
A7: LoG	The Laplacian of Gaussian of the original image was computed	1
A8: LN	The logarithmic derivative of the original image was computed	1
A9: SQUARED	The square derivative of the original image was computed	1
A10: SQRT	The square root derivative of the original image was computed	1

Abbreviations: A, Augmentation; UDWT, Undecimated Discrete Wavelet Transform; EXP, exponential; LN, logarithm; LoG, Laplacian of Gaussian; SQRT, square root; SQUARED, squared.

**Table 3:** Assembled datasets.

Dataset Name	Description
D1: All Augmentations	Includes all augmented images from each scenario
D2: UDWT + Spatial	Includes UDWT computed images and spatially augmented examples
D3: EXP + Spatial	Includes EXP computed images and spatially augmented examples
D4: LN + Spatial	Includes LN computed images and spatially augmented examples
D5: LoG + Spatial	Includes LoG computed images and spatially augmented examples
D6: Spatial Only	Includes only spatially augmented images
D7: SQRT + Spatial	Includes SQRT computed images and spatially augmented examples
D8: SQUARED + Spatial	Includes SQUARED computed images and spatially augmented examples

Abbreviations: UDWT, Undecimated Discrete Wavelet Transform; EXP, exponential; LN, logarithm; LoG, Laplacian of Gaussian; SQRT, square root; SQUARED, squared.

**Table 4:** Characteristics of the first data pool used for developing breast lesion detection functions.

Characteristics	Train	Validation	Test
Total number of patients	54	90	90
Total number of lesions	63	87	85
Subtypes			
Malignant	44 (70.0%)	35 (40.0%)	30 (35.0%)
Benign	19 (30.0%)	52 (60.0%)	59 (65.0%)

**Table 5:** Characteristics of the second data pool used for developing the breast lesion classification models.

Characteristics	Feature Selection Subset	Classification Subset	
	Total	Train	Test
Total number of patients	130	77	56
Total number of lesions	139	80	60
Subtypes			
Malignant	75 (54.0%)	40 (50.0%)	36 (60.0%)
Benign	64 (46.0%)	40 (50.0%)	24 (40.0%)

**Table 6:** The best breast lesion detection functions revealed in the validation step. The evaluation metrics (recall, precision, and F1-Score) was calculated with reference to the IoU score.

Dataset	Algorithm	IoU (Mean + STD)	Recall	Precision	F1-Score
D3: EXP + Spatial	Viola–Jones	0.3992 ± 0.0544	0.958	0.495	0.652
D4: LN + Spatial	YOLOv3	0.5362 ± 0.0640	0.824	0.805	0.814

**Table 7:** The best breast lesion detection functions revealed in the validation step. The evaluation metrics (recall, precision, and F1-Score) was calculated with reference to the LE score.

Dataset	Algorithm	LE (Mean + STD)	Recall	Precision	F1-Score
D3: EXP + Spatial	Viola–Jones	0.1208 ± 0.0146	0.970	0.699	0.813
D4: LN + Spatial	YOLOv3	0.1823 ± 0.0058	0.835	0.874	0.854

**Table 8:** The best breast lesion detection functions revealed in the test step. The evaluation metrics (recall, precision, and F1-Score) was calculated with reference to the IoU score.

Dataset	Algorithm	IoU (Mean + STD)	Recall	Precision	F1-Score
D1: All Augmentations	Viola–Jones	0.3986 ± 0.0540	0.959	0.500	0.657
D4: LN + Spatial	YOLOv3	0.5442 ± 0.0808	0.835	0.759	0.795

**Table 9:** The best breast lesion detection functions revealed in the test step. The evaluation metrics (recall, precision, and F1-Score) was calculated with reference to the LE score.

Dataset	Algorithm	LE (Mean + STD)	Recall	Precision	F1-Score
D1: All Augmentations	Viola–Jones	0.0959 ± 0.0162	0.972	0.734	0.836
D4: LN + Spatial	YOLOv3	0.1706 ± 0.0094	0.856	0.885	0.830

**Table 10:** Characteristics of Manual Segmentation, YOLOv3, and Viola–Jones breast lesion classification datasets.

Dataset	Number of Samples	Lambda	Number of Selected Features
Manual Segmentation	139	0.02	33
YOLOv3	139	0.01	51
Viola-Jones	139	0.02	41

**Table 11:** The precision metrics of the 3 best breast lesion classification models selected on the test groups of the Manual Segmentation, YOLOv3, and Viola–Jones Classification Subsets.

Method	Accuracy (%)	Sensitivity (%)	Specificity (%)
Weighted KNN (trained on Manual Segmentation dataset)	85.00	83.33	87.50
Ensemble Subspace KNN (trained on YOLOv3 dataset)	70.00	70.00	70.83
Median KNN (trained on Viola-Jones dataset)	61.67	61.11	62.50

**Table 12:** The statistical comparison of the 3 best breast lesion classification models with the Wilson Score Interval for estimation of lesion type discrimination probability.

AUROC Comparison	*p*-Value	z-Score
Ensemble Subspace KNN (trained on YOLOv3 dataset) and Weighted KNN (trained on Manual Segmentation dataset)	0.071	1.803
Median KNN (trained on Viola-Jones dataset) and Weighted KNN (trained on Manual Segmentation dataset)	0.002	−3.160

## Data Availability

The data presented in this study are available upon reasonable request from the corresponding author.

## Supplementary Materials

The following supporting information can be downloaded at: https://www.mdpi.com/article/10.3390/cancers14020277/s1, Table S1. The precision metrics (recall, precision, and F1-Score) calculated with reference to the IoU score for the best YOLOv3 detection models, trained with transfer learning by “fine tuning” and “freezing layers”, applied on the validation set. Table S2. The precision metrics (recall, precision, and F1-score) calculated with reference to the LE score for the best YOLOv3 detection models, trained with transfer learning by “fine tuning” and “freezing layers”, applied on the validation set. Table S3. The precision metrics (recall, precision, and F1-score) calculated with reference to the IoU score for the best YOLOv3 and Viola–Jones detection models applied on the validation set. Table S4. The precision metrics (recall, precision, and F1-score) calculated with reference to the LE score for the best YOLOv3 and Viola–Jones detection models applied on the validation set. Table S5. The precision metrics (recall, precision, and F1-score) calculated with reference to the IoU score for the best YOLOv3 and Viola–Jones detection models applied on the test set. Table S6. The precision metrics (recall, precision, and F1-score) calculated with reference to the LE score for the best YOLOv3 and Viola–Jones detection models applied on the test set. Table S7. The precision metrics (recall, precision, and F1-score) calculated with reference to the IoU score for the best YOLOv3 and Viola–Jones detection models applied on the second data pool. Table S8. The precision metrics (recall, precision, and F1-score) calculated with reference to the LE score for the best YOLOv3 and Viola–Jones detection models applied on the second data pool. Table S9. Radiomics features identified in the analysis of the Manual Segmentation dataset and their lambda score. Table S10. Radiomics features identified in the analysis of the YOLOv3 dataset and their lambda score. Table S11. Radiomics features identified in the analysis of the Viola–Jones dataset and their lambda score. Table S12. The precision metrics of 3 best breast lesion classification models trained with the 5-fold-cross-validation and 10-fold-cross-validation and 30%, 25%, and 20% holdout on training set of Manual Segmentation, YOLOv3, and Viola–Jones datasets Classification Subsets. Figure S1. Augmentation scenarios: Image pre-processing. Figure S2. Augmentation scenarios: Rotations. Figure S3. Augmentation scenarios: Translation. Figure S4. Augmentation scenarios: Flips. Figure S5. Augmentation scenarios: Shearing with respect to y-axis. Figure S6. Augmentation scenarios: Shearing with respect to x-axis. Figure S7. Labelling of positive and negative image samples for training Viola–Jones detection classifiers Figure S8. Recall-IoU and recall-LE characteristics resulting from the evaluation of the best breast lesion detection algorithm on the validation set. Figure S9. Recall-IoU and recall-LE characteristics resulting from the evaluation of the best breast lesion detection algorithm on the second data pool.
