# Supplementary material for: Influence of the Computer-Aided Decision Support System Design on Ultrasound-Based Breast Cancer Classification
Source: Cancers (Basel). 2022 Jan 6;14(2):277. doi: 10.3390/cancers14020277 (PMC8773857; doi:10.3390/cancers14020277)

**Supplementary Materials**

# Influence of the Computer-aided Decision Support System Design on Ultrasound-Based Breast Cancer Classification

## Supplementary Information

**Table S1.** The precision metrics (recall, precision, and F1-Score) calculated with reference to the IoU score for the best YOLOv3 detection models, trained with transfer learning by "fine tuning" and "freezing layers", applied on the validation set.

| Dataset | Method | IoU (mean + STD) | Recall | Precision | F1-Score |
|---|---|---|---|---|---|
| D1: All Augmentations | "fine tuning" | 0.5108 ± 0.0633 | 0.708 | 0.913 | 0.797 |
| | "freezing layers" | 0.4839 ± 0.0528 | 0.741 | 0.818 | 0.778 |
| D2: UDWT + Spatial | "fine tuning" | 0.5163 ± 0.0628 | 0.756 | 0.932 | 0.834 |
| | "freezing layers" | 0.3372 ± 0.0435 | 0.525 | 0.667 | 0.587 |
| D3: EXP + Spatial | "fine tuning" | 0.5087 ± 0.0596 | 0.753 | 0.905 | 0.822 |
| | "freezing layers" | 0.3383 ± 0.0429 | 0.536 | 0.804 | 0.643 |
| D4: LN + Spatial | "fine tuning" | 0.5362 ± 0.0640 | 0.824 | 0.805 | 0.814 |
| | "freezing layers" | 0.3929 ± 0.0485 | 0.567 | 0.895 | 0.694 |
| D5: LoG + Spatial | "fine tuning" | 0.5362 ± 0.0640 | 0.824 | 0.805 | 0.814 |
| | "freezing layers" | 0.3529 ± 0.0488 | 0.561 | 0.742 | 0.639 |
| D6: Spatial Only | "fine tuning" | 0.5085 ± 0.0647 | 0.688 | 0.970 | 0.805 |
| | "freezing layers" | 0.4123 ± 0.0503 | 0.632 | 0.797 | 0.705 |
| D7: SQRT + Spatial | "fine tuning" | 0.5403 ± 0.0722 | 0.773 | 0.883 | 0.824 |
| | "freezing layers" | 0.3489 ± 0.0468 | 0.554 | 0.730 | 0.630 |
| D8: SQUARED + Spatial | "fine tuning" | 0.5127 ± 0.0609 | 0.779 | 0.838 | 0.807 |
| | "freezing layers" | 0.3595 ± 0.0468 | 0.529 | 0.821 | 0.643 |

Abbreviations: UDWT, Undecimated Discrete Wavelet Transform; EXP, exponential; LN, logarithm; LoG, Laplacian of Gaussian; SQRT, square root; SQUARED, squared.

**Table S2.** The precision metrics (recall, precision, and F1-Score) calculated with reference to the LE score for the best YOLOv3 detection models, trained with transfer learning by 'fine tuning' and 'freezing layers', applied on the validation set.

| Dataset | Method | LE (mean + STD) | Recall | Precision | F1-Score |
|---|---|---|---|---|---|
| D1: All Augmentations | "fine tuning" | 0.2803 ± 0.0035 | 0.723 | 0.986 | 0.834 |
| | "freezing layers" | 0.2662 ± 0.0067 | 0.750 | 0.857 | 0.800 |
| D2: UDWT + Spatial | "fine tuning" | 0.2470 ± 0.0048 | 0.761 | 0.959 | 0.848 |
| | "freezing layers" | 0.4236 ± 0.0071 | 0.573 | 0.810 | 0.671 |
| D3: EXP + Spatial | "fine tuning" | 0.2499 ± 0.0051 | 0.763 | 0.959 | 0.850 |
| | "freezing layers" | 0.4369 ± 0.0058 | 0.557 | 0.875 | 0.681 |
| D4: LN + Spatial | "fine tuning" | 0.1823 ± 0.0058 | 0.835 | 0.874 | 0.854 |
| | "freezing layers" | 0.4222 ± 0.0042 | 0.576 | 0.930 | 0.711 |
| D5: LoG + Spatial | "fine tuning" | 0.1823 ± 0.0058 | 0.835 | 0.874 | 0.854 |
| | "freezing layers" | 0.4111 ± 0.0090 | 0.586 | 0.823 | 0.685 |
| D6: Spatial Only | "fine tuning" | 0.3122 ± 0.0044 | 0.691 | 0.985 | 0.813 |
| | "freezing layers" | 0.3514 ± 0.0071 | 0.648 | 0.855 | 0.738 |
| D7: SQRT + Spatial | "fine tuning" | 0.2273 ± 0.0063 | 0.783 | 0.935 | 0.852 |
| | "freezing layers" | 0.4054 ± 0.0057 | 0.589 | 0.841 | 0.693 |
| D8: SQUARED + Spatial | "fine tuning" | 0.2195 ± 0.0074 | 0.789 | 0.888 | 0.835 |
| | "freezing layers" | 0.4402 ± 0.0036 | 0.554 | 0.911 | 0.689 |

Abbreviations: UDWT, Undecimated Discrete Wavelet Transform; EXP, exponential; LN, logarithm; LoG, Laplacian of Gaussian; SQRT, square root; SQUARED, squared.

**Table S3.** The precision metrics (recall, precision, and F1-Score) calculated with reference to the IoU score for the best YOLOv3 and Viola-Jones detection models applied on the validation set. The metrics for the outstanding detection methods were underlined and bolded.

| Dataset | Method | IoU (mean + STD) | Recall | Precision | F1-Score |
|---|---|---|---|---|---|
| D1: All Augmentations | Viola-Jones | 0.4454 ± 0.0553 | 0.911 | 0.567 | 0.699 |
| | YOLOv3 | 0.5108 ± 0.0633 | 0.708 | 0.913 | 0.797 |
| D2: UDWT + Spatial | Viola-Jones | 0.4179 ± 0.0491 | 0.842 | 0.558 | 0.671 |
| | YOLOv3 | 0.5163 ± 0.0628 | 0.756 | 0.932 | 0.834 |
| **D3: EXP + Spatial** | **Viola-Jones** | **0.3992 ± 0.0544** | **0.958** | **0.495** | **0.652** |
| | YOLOv3 | 0.5087 ± 0.0596 | 0.753 | 0.905 | 0.822 |
| **D4: LN + Spatial** | Viola-Jones | 0.4208 ± 0.0571 | 0.943 | 0.543 | 0.690 |
| | **YOLOv3** | **0.5362 ± 0.0640** | **0.824** | **0.805** | **0.814** |
| D5: LoG + Spatial | Viola-Jones | 0.3677 ± 0.0493 | 0.849 | 0.517 | 0.643 |
| | YOLOv3 | 0.5362 ± 0.0640 | 0.824 | 0.805 | 0.814 |
| D6: Spatial Only | Viola-Jones | 0.4066 ± 0.0519 | 0.939 | 0.500 | 0.652 |
| | YOLOv3 | 0.5085 ± 0.0647 | 0.688 | 0.970 | 0.805 |
| D7: SQRT + Spatial | Viola-Jones | 0.3801 ± 0.0497 | 0.797 | 0.566 | 0.662 |
| | YOLOv3 | 0.5403 ± 0.0722 | 0.773 | 0.883 | 0.824 |
| D8: SQUARED + Spatial | Viola-Jones | 0.3990 ± 0.052 | 0.922 | 0.516 | 0.662 |
| | YOLOv3 | 0.5127 ± 0.0609 | 0.779 | 0.838 | 0.807 |

Abbreviations: UDWT, Undecimated Discrete Wavelet Transform; EXP, exponential; LN, logarithm; LoG, Laplacian of Gaussian; SQRT, square root; SQUARED, squared.

**Table S4.** The precision metrics (recall, precision, and F1-Score) calculated with reference to the LE score for the best YOLOv3 and Viola-Jones detection models applied on the validation set. The metrics for the outstanding detection methods were underlined and bolded.

| Dataset | Method | LE (mean + STD) | Recall | Precision | F1-Score |
|---|---|---|---|---|---|
| D1: All Augmentations | Viola-Jones | 0.1389 ± 0.0132 | 0.932 | 0.767 | 0.841 |
| | YOLOv3 | 0.2803 ± 0.0035 | 0.723 | 0.986 | 0.834 |
| D2: UDWT + Spatial | Viola-Jones | 0.1779 ± 0.0120 | 0.880 | 0.767 | 0.820 |
| | YOLOv3 | 0.2470 ± 0.0048 | 0.761 | 0.959 | 0.848 |
| **D3: EXP + Spatial** | **Viola-Jones** | **0.1208 ± 0.0146** | **0.970** | **0.699** | **0.813** |
| | YOLOv3 | 0.2499 ± 0.0051 | 0.763 | 0.959 | 0.850 |
| **D4: LN + Spatial** | Viola-Jones | 0.1144 ± 0.0139 | 0.957 | 0.728 | 0.827 |
| | **YOLOv3** | **0.1823 ± 0.0058** | **0.835** | **0.874** | **0.854** |
| D5: LoG + Spatial | Viola-Jones | 0.1886 ± 0.0127 | 0.882 | 0.690 | 0.774 |
| | YOLOv3 | 0.1823 ± 0.0058 | 0.835 | 0.874 | 0.854 |
| D6: Spatial Only | Viola-Jones | 0.1236 ± 0.0126 | 0.958 | 0.739 | 0.834 |
| | YOLOv3 | 0.3122 ± 0.0044 | 0.691 | 0.985 | 0.813 |
| D7: SQRT + Spatial | Viola-Jones | 0.1532 ± 0.0137 | 0.833 | 0.723 | 0.774 |
| | YOLOv3 | 0.2273 ± 0.0063 | 0.783 | 0.935 | 0.852 |
| D8: SQUARED + Spatial | Viola-Jones | 0.1369 ± 0.0142 | 0.942 | 0.714 | 0.813 |
| | YOLOv3 | 0.2195 ± 0.0074 | 0.789 | 0.888 | 0.835 |

Abbreviations: UDWT, Undecimated Discrete Wavelet Transform; EXP, exponential; LN, logarithm; LoG, Laplacian of Gaussian; SQRT, square root; SQUARED, squared.

**Table S5.** The precision metrics (recall, precision, and F1-Score) calculated with reference to the IoU score for the best YOLOv3 and Viola-Jones detection models applied on **the test set**. The metrics for the outstanding detection methods were underlined and bolded.

| Dataset | Method | IoU (mean + STD) | Recall | Precision | F1-Score |
|---|---|---|---|---|---|
| **D1: All Augmentations** | **Viola-Jones** | **0.3986 ± 0.0540** | **0.959** | **0.500** | **0.657** |
| | YOLOv3 | 0.4390 ± 0.0636 | 0.626 | 0.919 | 0.745 |
| D2: UDWT + Spatial | Viola-Jones | 0.3906 ± 0.0458 | 0.868 | 0.517 | 0.648 |
| | YOLOv3 | 0.5044 ± 0.0638 | 0.733 | 0.917 | 0.815 |
| D3: EXP + Spatial | Viola-Jones | 0.3426 ± 0.0477 | 0.944 | 0.362 | 0.523 |
| | YOLOv3 | 0.3998 ± 0.0569 | 0.612 | 0.788 | 0.689 |
| **D4: LN + Spatial** | Viola-Jones | 0.3555 ± 0.0486 | 0.902 | 0.402 | 0.556 |
| | **YOLOv3** | **0.5442 ± 0.0808** | **0.835** | **0.759** | **0.795** |
| D5: LoG + Spatial | Viola-Jones | 0.3687 ± 0.0465 | 0.872 | 0.456 | 0.599 |
| | YOLOv3 | 0.5329 ± 0.0785 | 0.823 | 0.756 | 0.788 |
| D6: Spatial Only | Viola-Jones | 0.3795 ± 0.0542 | 0.860 | 0.483 | 0.619 |
| | YOLOv3 | 0.5003 ± 0.0586 | 0.696 | 0.914 | 0.790 |
| D7: SQRT + Spatial | Viola-Jones | 0.3602 ± 0.0461 | 0.843 | 0.489 | 0.619 |
| | YOLOv3 | 0.4862 ± 0.0710 | 0.713 | 0.873 | 0.785 |
| D8: SQUARED + Spatial | Viola-Jones | 0.3447 ± 0.0461 | 0.854 | 0.389 | 0.534 |
| | YOLOv3 | 0.5152 ± 0.0640 | 0.805 | 0.833 | 0.819 |

Abbreviations: UDWT, Undecimated Discrete Wavelet Transform; EXP, exponential; LN, logarithm; LoG, Laplacian of Gaussian; SQRT, square root; SQUARED, squared.

**Table S6.** The precision metrics (recall, precision, and F1-Score) calculated with reference to the LE score for the best YOLOv3 and Viola-Jones detection models applied on **the test set**. The metrics for the outstanding detection methods were underlined and bolded.

| Dataset | Method | LE (mean + STD) | Recall | Precision | F1-Score |
|---|---|---|---|---|---|
| **D1: All Augmentations** | **Viola-Jones** | **0.0959 ± 0.0162** | **0.972** | **0.734** | **0.836** |
| | YOLOv3 | 0.3741 ± 0.0071 | 0.630 | 0.935 | 0.753 |
| D2: UDWT + Spatial | Viola-Jones | 0.1560 ± 0.0126 | 0.908 | 0.775 | 0.836 |
| | YOLOv3 | 0.2698 ± 0.0047 | 0.739 | 0.944 | 0.829 |
| D3: EXP + Spatial | Viola-Jones | 0.1066 ± 0.0151 | 0.969 | 0.660 | 0.785 |
| | YOLOv3 | 0.3667 ± 0.0086 | 0.637 | 0.879 | 0.739 |
| **D4: LN + Spatial** | Viola-Jones | 0.1205 ± 0.0145 | 0.944 | 0.739 | 0.829 |
| | **YOLOv3** | **0.1706 ± 0.0094** | **0.856** | **0.885** | **0.830** |
| D5: LoG + Spatial | Viola-Jones | 0.1420 ± 0.0153 | 0.915 | 0.722 | 0.807 |
| | YOLOv3 | 0.1801 ± 0.0090 | 0.844 | 0.884 | 0.864 |
| D6: Spatial Only | Viola-Jones | 0.1385 ± 0.0134 | 0.907 | 0.764 | 0.829 |
| | YOLOv3 | 0.3020 ± 0.0048 | 0.702 | 0.943 | 0.805 |
| D7: SQRT + Spatial | Viola-Jones | 0.1533 ± 0.0136 | 0.889 | 0.727 | 0.800 |
| | YOLOv3 | 0.2815 ± 0.0079 | 0.725 | 0.930 | 0.815 |
| D8: SQUARED + Spatial | Viola-Jones | 0.1430 ± 0.0150 | 0.913 | 0.700 | 0.792 |
| | YOLOv3 | 0.2088 ± 0.0094 | 0.811 | 0.869 | 0.839 |

Abbreviations: UDWT, Undecimated Discrete Wavelet Transform; EXP, exponential; LN, logarithm; LoG, Laplacian of Gaussian; SQRT, square root; SQUARED, squared.

**Table S7.** The precision metrics (recall, precision, and F1-Score) calculated with reference to the IoU score for the best YOLOv3 and Viola-Jones detection models applied on the second data pool. The metrics for the outstanding detection methods were underlined and bolded.

| Dataset | Method | IoU (mean + STD) | Recall | Precision | F1-Score |
|---|---|---|---|---|---|
| D1: All Augmentations | Viola-Jones | 0.2825 ± 0.0512 | 0.817 | 0.331 | 0.471 |
| | YOLOv3 | 0.3485 ± 0.0512 | 0.540 | 0.753 | 0.629 |
| D2: UDWT + Spatial | Viola-Jones | 0.2631± 0.0470 | 0.802 | 0.316 | 0.454 |
| | YOLOv3 | 0.3819 ± 0.0594 | 0.609 | 0.729 | 0.664 |
| D3: EXP + Spatial | Viola-Jones | 0.2443 ± 0.0493 | 0.873 | 0.232 | 0.367 |
| | YOLOv3 | 0.3520 ± 0.0511 | 0.547 | 0.792 | 0.647 |
| **D4: LN + Spatial** | **Viola-Jones** | **0.2654 ± 0.0560** | **0.896** | **0.257** | **0.400** |
| | **YOLOv3** | **0.4247 ± 0.0609** | **0.729** | **0.664** | **0.695** |
| D5: LoG + Spatial | Viola-Jones | 0.2681 ± 0.0565 | 0.855 | 0.269 | 0.409 |
| | YOLOv3 | 0.4247 ± 0.0609 | 0.729 | 0.664 | 0.695 |
| D6: Spatial Only | Viola-Jones | 0.2886 ± 0.0545 | 0.845 | 0.335 | 0.479 |
| | YOLOv3 | 0.3241 ± 0.0436 | 0.479 | 0.758 | 0.587 |
| D7: SQRT + Spatial | Viola-Jones | 0.2753 ± 0.0526 | 0.806 | 0.341 | 0.479 |
| | YOLOv3 | 0.3885 ± 0.0595 | 0.591 | 0.761 | 0.665 |
| D8: SQUARED + Spatial | Viola-Jones | 0.2807 ± 0.0553 | 0.854 | 0.313 | 0.458 |
| | YOLOv3 | 0.4273 ± 0.0689 | 0.723 | 0.719 | 0.721 |

Abbreviations: UDWT, Undecimated Discrete Wavelet Transform; EXP, exponential; LN, logarithm; LoG, Laplacian of Gaussian; SQRT, square root; SQUARED, squared.

**Table S8.** The precision metrics (recall, precision, and F1-Score) calculated with reference to the LE score for the best YOLOv3 and Viola-Jones detection models applied on the second data pool. The metrics for the outstanding detection methods were underlined and bolded.

| Dataset | Method | LE (mean + STD) | Recall | Precision | F1-Score |
|---|---|---|---|---|---|
| D1: All Augmentations | Viola-Jones | 0.2428 ± 0.0392 | 0.886 | 0.572 | 0.695 |
| | YOLOv3 | 0.4125 ± 0.0085 | 0.584 | 0.900 | 0.708 |
| D2: UDWT + Spatial | Viola-Jones | 0.2736 ± 0.0396 | 0.869 | 0.520 | 0.650 |
| | YOLOv3 | 0.3471 ± 0.0116 | 0.656 | 0.896 | 0.758 |
| D3: EXP + Spatial | Viola-Jones | 0.2426 ± 0.0384 | 0.934 | 0.479 | 0.634 |
| | YOLOv3 | 0.4181 ± 0.0083 | 0.583 | 0.917 | 0.713 |
| **D4: LN + Spatial** | **Viola-Jones** | **0.2187 ± 0.0378** | **0.948** | **0.549** | **0.695** |
| | **YOLOv3** | **0.2491 ± 0.0168** | **0.769** | **0.821** | **0.794** |
| D5: LoG + Spatial | Viola-Jones | 0.2310 ± 0.0387 | 0.917 | 0.504 | 0.650 |
| | YOLOv3 | 0.2491 ± 0.0168 | 0.769 | 0.821 | 0.794 |
| D6: Spatial Only | Viola-Jones | 0.2285 ± 0.0376 | 0.901 | 0.562 | 0.692 |
| | YOLOv3 | 0.4760 ± 0.0085 | 0.512 | 0.863 | 0.642 |
| D7: SQRT + Spatial | Viola-Jones | 0.2628 ± 0.0363 | 0.873 | 0.565 | 0.686 |
| | YOLOv3 | 0.3635 ± 0.0090 | 0.641 | 0.940 | 0.762 |
| D8: SQUARED + Spatial | Viola-Jones | 0.2326 ± 0.0366 | 0.912 | 0.553 | 0.689 |
| | YOLOv3 | 0.2565 ± 0.0151 | 0.760 | 0.873 | 0.813 |

Abbreviations: UDWT, Undecimated Discrete Wavelet Transform; EXP, exponential; LN, logarithm; LoG, Laplacian of Gaussian; SQRT, square root; SQUARED, squared.

**Table S9.** Radiomics features identified in the analysis of the Manual Segmentation dataset and their lambda score.

| Feature Name | Lambda |
|---|---|
| original_glcm_Imc2 | 0.011928 |
| original_glszm_LowGrayLevelZoneEmphasis | 0.023903 |
| exponential_glcm_Imc1 | 0.007132 |
| exponential_glrlm_GrayLevelNonUniformity | 0.037015 |
| exponential_glrlm_HighGrayLevelRunEmphasis | -0.01403 |
| exponential_glrlm_RunEntropy | 0.048686 |
| exponential_glszm_SmallAreaLowGrayLevelEmphasis | 0.004808 |
| gradient_firstorder_Skewness | -0.09664 |
| gradient_glrlm_RunEntropy | 0.130352 |
| gradient_glrlm_ShortRunLowGrayLevelEmphasis | -0.06101 |
| logarithm_glcm_Contrast | -0.03666 |
| logarithm_glcm_Idmn | 0.020441 |
| logarithm_glcm_Imc2 | 0.019052 |
| logarithm_glrlm_RunVariance | -0.023 |
| logarithm_glszm_GrayLevelNonUniformityNormalized | -0.04194 |
| squareroot_glrlm_LongRunHighGrayLevelEmphasis | -0.01728 |
| squareroot_glszm_LargeAreaHighGrayLevelEmphasis | -0.01028 |
| squareroot_glszm_SmallAreaHighGrayLevelEmphasis | -0.00084 |
| wavelet-LH_firstorder_Mean | -0.01353 |
| wavelet-LH_firstorder_Skewness | -0.04576 |
| wavelet-LH_glcm_Imc1 | 0.026884 |
| wavelet-LH_glszm_SmallAreaEmphasis | -0.11354 |
| wavelet-LH_ngtdm_Contrast | 0.012435 |
| wavelet-HL_firstorder_Energy | 0.020368 |
| wavelet-HL_firstorder_TotalEnergy | 1.28E-17 |
| wavelet-HL_glrlm_GrayLevelNonUniformityNormalized | -0.02213 |
| wavelet-HL_glrlm_ShortRunEmphasis | 0.081554 |
| wavelet-HL_glszm_SmallAreaEmphasis | 0.036429 |
| wavelet-HH_glszm_GrayLevelVariance | 0.006782 |
| wavelet-HH_glszm_LowGrayLevelZoneEmphasis | 0.00159 |
| wavelet-HH_glszm_SmallAreaEmphasis | 0.003901 |
| wavelet-HH_glszm_SmallAreaLowGrayLevelEmphasis | 0.038128 |
| wavelet-LL_glszm_SmallAreaLowGrayLevelEmphasis | -0.01804 |

**Table S10.** Radiomics features identified in the analysis of the YOLOv3 dataset and their lambda score.

| Feature name | Lambda |
| --- | --- |
| original_gldm_HighGrayLevelEmphasis | -0.0307695 |
| original_glrlm_LongRunHighGrayLevelEmphasis | -0.00035511 |
| original_glrlm_ShortRunLowGrayLevelEmphasis | -0.0922137 |
| original_glszm_LargeAreaLowGrayLevelEmphasis | 0.0107439 |
| original_glszm_SmallAreaEmphasis | 0.0265081 |
| exponential_firstorder_Kurtosis | 0.00206107 |
| exponential_firstorder_Median | -0.0502144 |
| exponential_glcm_Idmn | 0.0172652 |
| exponential_glcm_JointEnergy | 0.0355724 |
| exponential_gldm_LargeDependenceHighGrayLevelEmphasis | -0.0115515 |
| exponential_glszm_GrayLevelVariance | 0.120248 |
| exponential_ngtdm_Coarseness | -0.0320669 |
| gradient_glcm_Correlation | -0.0251904 |
| gradient_glrlm_RunLengthNonUniformityNormalized | 0.136493 |
| lbp-2D_firstorder_10Percentile | -0.054692 |
| lbp-2D_firstorder_90Percentile | -0.0211308 |
| lbp-2D_firstorder_RobustMeanAbsoluteDeviation | -0.00169874 |
| lbp-2D_glrlm_RunEntropy | 0.0799155 |
| logarithm_glcm_Correlation | 0.00244712 |
| logarithm_glrlm_ShortRunLowGrayLevelEmphasis | -0.0416502 |
| logarithm_glszm_LowGrayLevelZoneEmphasis | -0.0295585 |
| logarithm_glszm_SizeZoneNonUniformity | 0.0178617 |
| square_glszm_LowGrayLevelZoneEmphasis | 0.0420735 |
| squareroot_firstorder_Kurtosis | 0.0409406 |
| squareroot_glszm_HighGrayLevelZoneEmphasis | -0.0399423 |
| squareroot_glszm_SmallAreaLowGrayLevelEmphasis | 0.022961 |
| squareroot_ngtdm_Busyness | 0.00390109 |
| wavelet-LH_firstorder_Kurtosis | -0.0265017 |
| wavelet-LH_gldm_DependenceVariance | -0.0843548 |
| wavelet-LH_gldm_SmallDependenceEmphasis | -0.0927388 |
| wavelet-LH_glszm_SmallAreaLowGrayLevelEmphasis | -0.0680015 |
| wavelet-LH_glszm_ZoneEntropy | 0.0303013 |
| wavelet-LH_ngtdm_Contrast | 0.0800897 |
| wavelet-HL_firstorder_Mean | 0.000534665 |
| wavelet-HL_firstorder_Median | 0.0620706 |
| wavelet-HL_firstorder_Skewness | 0.0139484 |
| wavelet-HL_gldm_DependenceEntropy | 0.0255289 |
| wavelet-HL_glrlm_LongRunLowGrayLevelEmphasis | -0.0709579 |
| wavelet-HL_glrlm_ShortRunEmphasis | 0.125298 |
| wavelet-HL_glszm_LargeAreaLowGrayLevelEmphasis | 0.00545148 |
| wavelet-HL_glszm_LowGrayLevelZoneEmphasis | -0.0263626 |
| wavelet-HH_firstorder_Median | 0.0439149 |

| | |
|---|---|
| wavelet-HH_firstorder_Skewness | 0.0837893 |
| wavelet-HH_glcm_Imc2 | -0.206597 |
| wavelet-HH_glrlm_RunLengthNonUniformityNormalized | -0.0275014 |
| wavelet-HH_glrlm_ShortRunEmphasis | -0.0268027 |
| wavelet-HH_glszm_LargeAreaHighGrayLevelEmphasis | -0.088222 |
| wavelet-HH_glszm_SizeZoneNonUniformityNormalized | 0.0776143 |
| wavelet-LL_glszm_GrayLevelVariance | 0.0112277 |
| wavelet-LL_glszm_SmallAreaLowGrayLevelEmphasis | -0.0282413 |
| wavelet-LL_ngtdm_Busyness | 0.0591829 |

**Table S11.** Radiomics features identified in the analysis of the Viola-Jones dataset and their lambda score.

| Feature name | Lambda |
|---|---|
| original_glrlm_LongRunHighGrayLevelEmphasis | -0.02305 |
| original_glszm_LargeAreaLowGrayLevelEmphasis | 0.022992 |
| original_glszm_LowGrayLevelZoneEmphasis | 0.044806 |
| exponential_glcm_ClusterShade | 0.023199 |
| exponential_glrlm_LongRunLowGrayLevelEmphasis | 0.011298 |
| exponential_glrlm_RunLengthNonUniformityNormalized | 0.010645 |
| exponential_glrlm_RunVariance | 0.022613 |
| exponential_glrlm_ShortRunLowGrayLevelEmphasis | -0.01934 |
| exponential_glszm_ZonePercentage | 0.017238 |
| exponential_glszm_ZoneVariance | 0.002615 |
| exponential_ngtdm_Strength | 0.023735 |
| gradient_firstorder_10Percentile | 0.005552 |
| gradient_firstorder_Kurtosis | 0.047964 |
| gradient_firstorder_Minimum | 0.010106 |
| gradient_glrlm_RunLengthNonUniformityNormalized | 0.152847 |
| gradient_glszm_LargeAreaEmphasis | -0.01393 |
| gradient_glszm_SmallAreaEmphasis | -0.04935 |
| logarithm_firstorder_Kurtosis | 0.025563 |
| logarithm_firstorder_Skewness | -0.00622 |
| logarithm_glszm_SmallAreaLowGrayLevelEmphasis | -0.05485 |
| logarithm_ngtdm_Strength | -0.02842 |
| square_ngtdm_Coarseness | 0.004001 |
| squareroot_glcm_ClusterShade | -0.01944 |
| squareroot_glrlm_ShortRunLowGrayLevelEmphasis | -0.01013 |
| squareroot_glszm_LowGrayLevelZoneEmphasis | 0.023115 |
| squareroot_ngtdm_Contrast | -0.02298 |
| squareroot_ngtdm_Strength | -0.05153 |
| wavelet-LH_firstorder_Skewness | 0.026742 |
| wavelet-LH_glcm_ClusterProminence | -0.05829 |
| wavelet-LH_glcm_Idmn | -0.00129 |
| wavelet-LH_glcm_Imc1 | 0.02695 |
| wavelet-LH_glszm_SmallAreaEmphasis | -0.02663 |
| wavelet-HL_firstorder_Skewness | -0.05648 |
| wavelet-HL_gldm_DependenceNonUniformityNormalized | -0.03142 |
| wavelet-HL_glszm_LargeAreaHighGrayLevelEmphasis | 0.010999 |
| wavelet-HH_firstorder_Skewness | 0.006642 |
| wavelet-HH_glcm_ClusterShade | 0.003819 |
| wavelet-HH_glcm_Imc2 | -0.03124 |
| wavelet-HH_gldm_DependenceNonUniformityNormalized | -0.03556 |
| wavelet-HH_glszm_SizeZoneNonUniformityNormalized | 0.065 |
| wavelet-HH_glszm_SmallAreaLowGrayLevelEmphasis | 0.01799 |

**Table S12.** The precision metrics of 3 best breast lesion classification models trained with the 5-fold-cross-validation and 10-fold-cross-validation and 30%, 25%, and 20% holdout on training set of Manual Segmentation, YOLOv3, and Viola-Jones datasets Classification Subsets. The selected algorithms were evaluated with respect to their AUROC, accuracy, sensitivity, and specificity. The metrics for the outstanding classification model obtained for Manual Segmentation, YOLOv3, and Viola-Jones dataset were underlined and bolded.

| **Dataset** | Training | Algorithm | AUROC | Accuracy(%) | Sensitivity(%) | Specificity(%) |
|---|---|---|---|---|---|---|
| **Manual Segmentation** | 5-fold-cross-validation | Linear SVM | 0.89 | 83.33 | 80.56 | 87.50 |
| | | **Weighted KNN** | **0.94** | **85.00** | **83.33** | **87.50** |
| | | Ensemble Subspace Discriminant | 0.91 | 83.33 | 86.11 | 79.17 |
| | 10-fold-cross-validation | Linear SVM | 0.89 | 83.33 | 80.56 | 87.50 |
| | | **Weighted KNN** | **0.94** | **85.00** | **83.33** | **87.50** |
| | | Ensemble Subspace Discriminant | 0.91 | 81.67 | 83.33 | 79.17 |
| | 30% holdout validation | Linear SVM | 0.89 | 83.33 | 80.56 | 87.50 |
| | | **Weighted KNN** | **0.94** | **85.00** | **83.33** | **87.50** |
| | | Ensemble Subspace Discriminant | 0.90 | 81.67 | 83.33 | 79.17 |
| | 25% holdout validation | Cubic SVM | 0.91 | 85.00 | 83.33 | 87.50 |
| | | Fine KNN | 0.82 | 81.67 | 80.56 | 83.33 |
| | | Ensemble Subspace Discriminant | 0.92 | 83.33 | 83.33 | 83.33 |
| | 20% holdout validation | Linear SVM | 0.89 | 83.33 | 80.56 | 87.50 |
| | | Weighted KNN | 0.91 | 85.00 | 83.33 | 87.50 |
| | | Ensemble Subspace Discriminant | 0.91 | 81.67 | 83.33 | 79.17 |
| **YOLOv3** | 5-fold-cross-validation | Linear SVM | 0.74 | 66.67 | 63.89 | 70.83 |
| | | **Ensemble Subspace KNN** | **0.81** | **70.00** | **70.00** | **70.83** |
| | | Ensemble Bagged Trees | 0.77 | 66.67 | 66.67 | 66.67 |
| | 10-fold-cross-validation | Linear SVM | 0.74 | 66.67 | 63.89 | 70.83 |
| | | Ensemble Subspace KNN | 0.81 | 71.67 | 75.00 | 66.67 |
| | | Ensemble Bagged Trees | 0.77 | 71.67 | 72.22 | 70.83 |
| | 30% holdout validation | Coarse Gaussian SVM | 0.73 | 73.33 | 83.33 | 58.33 |
| | | Fine KNN | 0.73 | 73.33 | 75.00 | 70.83 |

| | | | | | | |
|---|---|---|---|---|---|---|
| | | Ensemble Subspace Discriminant | 0.72 | 63.33 | 63.89 | 62.50 |
| | 25% holdout validation | Quadratic SVM | 0.79 | 40.00 | 00.00 | 100.0 |
| | | Ensemble Bagged Trees | 0.79 | 71.67 | 80.56 | 58.33 |
| | | Ensemble Subspace Discriminant | 0.74 | 66.67 | 66.67 | 66.67 |
| | 20% holdout validation | Ensemble Bagged Trees | 0.78 | 71.67 | 75.00 | 66.67 |
| | | Linear SVM | 0.74 | 66.67 | 63.89 | 70.83 |
| | | Weighted KNN | 0.75 | 66.67 | 58.33 | 79.17 |
| | 5-fold-cross-validation | Cosine KNN | 0.67 | 55.00 | 52.78 | 58.33 |
| | | **Median KNN** | **0.70** | **61.67** | **61.11** | **62.50** |
| | | Ensemble Subspace Discriminant | 0.66 | 60.00 | 69.44 | 45.83 |
| | 10-fold-cross-validation | Cosine KNN | 0.67 | 55.00 | 52.78 | 58.33 |
| | | Linear SVM | 0.33 | 40.00 | 00.00 | 100.00 |
| | | Ensemble Subspace Discriminant | 0.66 | 58.33 | 66.67 | 45.83 |
| **Viola-Jones** | 30% holdout validation | Coarse Gaussian SVM | 0.65 | 68.33 | 86.11 | 41.67 |
| | | Fine Tree | 0.59 | 61.67 | 61.11 | 62.50 |
| | | Linear SVM | 0.33 | 40.00 | 00.00 | 100.00 |
| | 25% holdout validation | GaussianNaïve Bayes | 0.59 | 41.67 | 11.11 | 87.50 |
| | | Kernel Naïve Bayes | 0.59 | 48.33 | 44.44 | 54.17 |
| | | Linear SVM | 0.33 | 40.00 | 00.00 | 100.00 |
| | 20% holdout validation | Linear SVM | 0.33 | 40.00 | 00.00 | 100.00 |
| | | Kernel Naïve Bayes | 0.59 | 48.33 | 44.44 | 54.17 |
| | | Median KNN | 0.70 | 61.67 | 61.11 | 62.50 |

**Figure S1.** Augmentation scenarios: Image pre-processing. (A) Original image; (B) Image decomposed with UDWT (HH); (C) Image decomposed with UDWT (HL); (D) Image decomposed with UDWT (LH); (E) Image decomposed with UDWT (LL); (F) Exponential derivative of the original image; (G) Logarithmic derivative of the original image; (H) Laplacian of Gaussian of the original image; (I) Squared derivative of the original image; (J) Square root derivative of the original image.

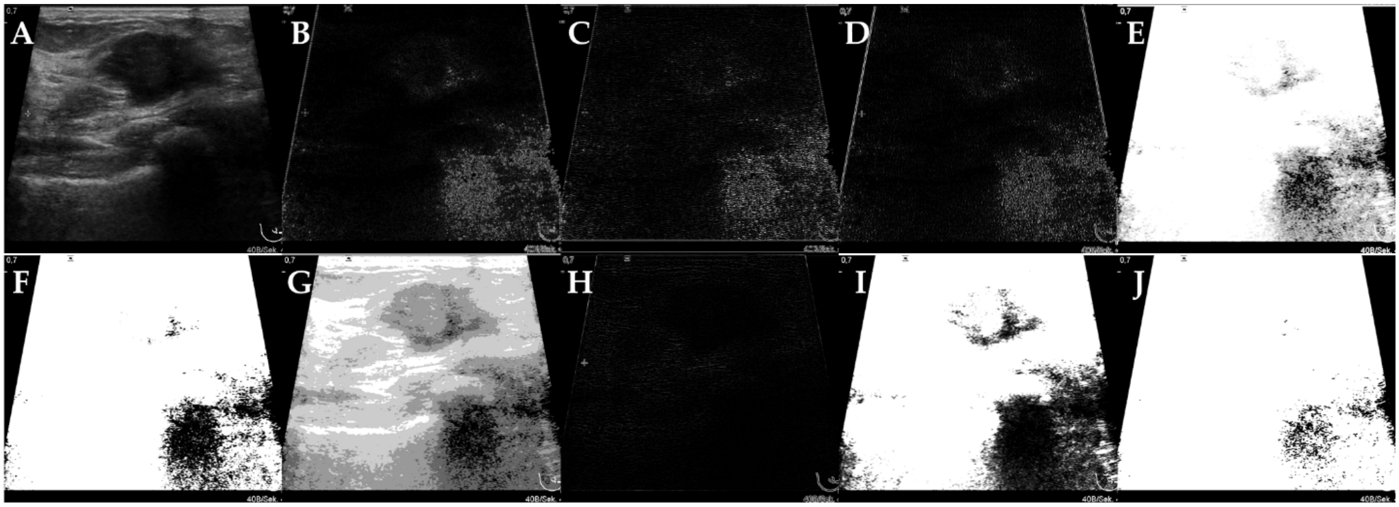


**Figure S2.** Augmentation scenarios: Rotations. Original image rotated: (A-i) Clockwise by 45º; (A-ii) Clockwise by 90º; (A-iii) Clockwise by 135º; (B-i) Counterclockwise by 45º; (B-ii) Counterclockwise by 90º; (B-iii) Counterclockwise by 135º.

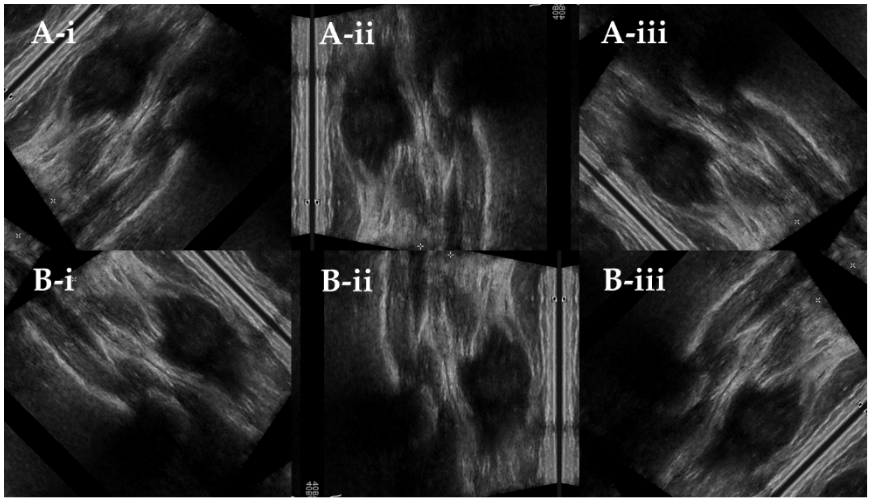

**Figure S3.** Augmentation scenarios: Translation. Original image translated: (i) Right (x-axis) and Down (y-axis); (ii) Right (x-axis) and Up (y-axis); (iii) Left (x-axis) and Down (y-axis); (iv) Left (x-axis) and Up (y-axis).

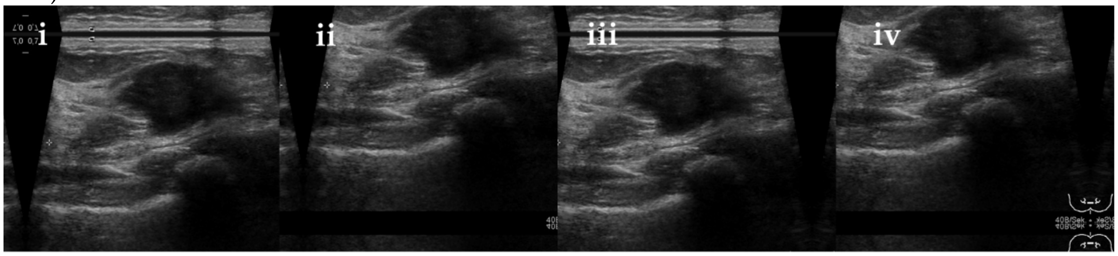


**Figure S4**. Augmentation scenarios: Flips. Original image flipped about: (A-i) Its origin; (A-ii) X-axis; (A-iii) Y-axis.

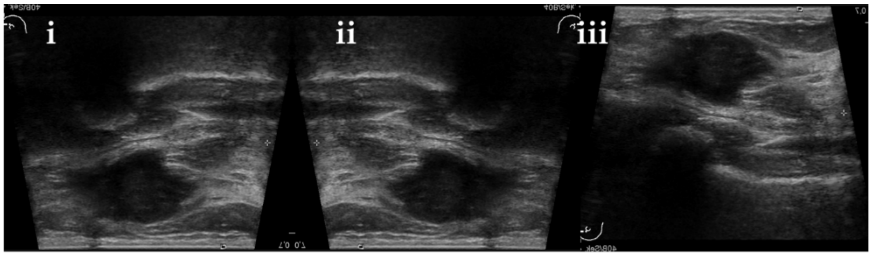

**Figure S5.** Augmentation scenarios: Shearing with respect to y-axis. Original image sheared right by: (A-i) 5º; (A-ii) 10º; (A-iii) 15º; (A-iv) 20º, (A-v) 25º; (A-vi) 30º. Original image flipped about its origin and sheared right by: (B-i) 5º; (B-ii) 10º; (B-iii) 15º; (B-iv) 20º, (B-v) 25º; (B-vi) 30º. Original image flipped about x-axis and sheared right by: (C-i) 5º; (C-ii) 10º; (C-iii) 15º; (C-iv) 20º, (C-v) 25º; (C-vi) 30º. Original image flipped about y-axis and sheared right by: (D-i) 5º; (D-ii) 10º; (D-iii) 15º; (D-iv) 20º, (D-v) 25º; (D-vi) 30º. Original image sheared left by: (E-i) 5º; (E-ii) 10º; (E-iii) 15º; (E-iv) 20º, (E-v) 25º; (E-vi) 30º. Original image flipped about its origin and sheared left by: (F-i) 5º; (F-ii) 10º; (F-iii) 15º; (F-iv) 20º, (F-v) 25º; (F-vi) 30º. Original image flipped about x-axis and sheared left by: (G-i) 5º; (G-ii) 10º; (G-iii) 15º; (G-iv) 20º, (G-v) 25º; (G-vi) 30º. Original image flipped about y-axis and sheared left by: (H-i) 5º; (H-ii) 10º; (H-iii) 15º; (H-iv) 20º, (H-v) 25º; (H-vi) 30º.

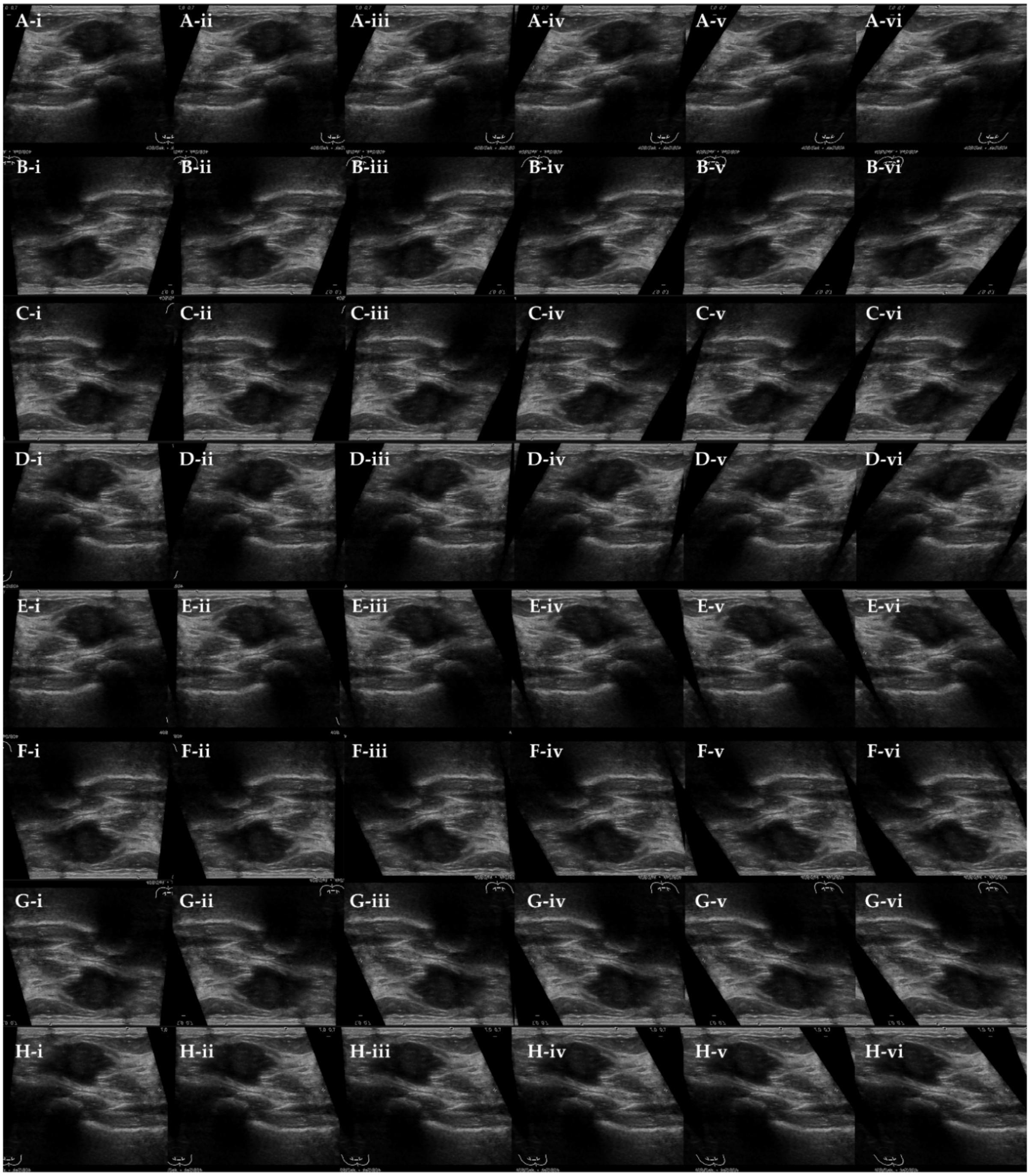

**Figure S6.** Augmentation scenarios: Shearing with respect to x-axis. Original image sheared down by: (A-i) 5º; (A-ii) 10º; (A-iii) 15º; (A-iv) 20º, (A-v) 25º; (A-vi) 30º. Original image flipped about its origin and sheared down by: (B-i) 5º; (B-ii) 10º; (B-iii) 15º; (B-iv) 20º, (B-v) 25º; (B-vi) 30º. Original image flipped about x-axis and sheared down by: (C-i) 5º; (C-ii) 10º; (C-iii) 15º; (C-iv) 20º, (C-v) 25º; (C-vi) 30º. Original image flipped about y-axis and sheared down by: (D-i) 5º; (D-ii) 10º; (D-iii) 15º; (D-iv) 20º, (D-v) 25º; (D-vi) 30º. Original image sheared up by: (E-i) 5º; (E-ii) 10º; (E-iii) 15º; (E-iv) 20º, (E-v) 25º; (E-vi) 30º. Original image flipped about its origin and sheared up by: (F-i) 5º; (F-ii) 10º; (F-iii) 15º; (F-iv) 20º, (F-v) 25º; (F-vi) 30º. Original image flipped about x-axis and sheared up by: (G-i) 5º; (G-ii) 10º; (G-iii) 15º; (G-iv) 20º, (G-v) 25º; Original image flipped about y-axis and sheared up by: (H-i) 5º; (H-ii) 10º; (H-iii) 15º; (H-iv) 20º, (H-v) 25º; (H-vi) 30º.

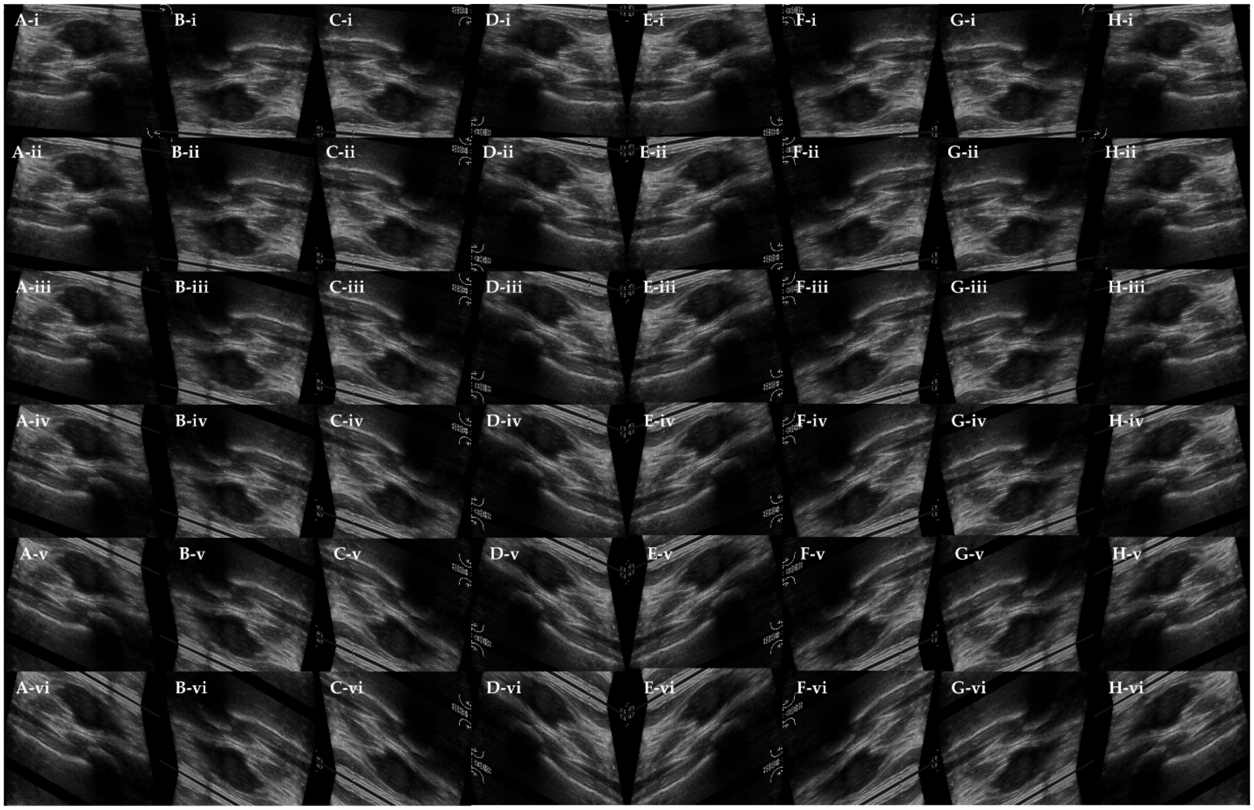

**Figure S7.** Labeling of positive and negative image samples for training Viola-Jones detection classifiers.

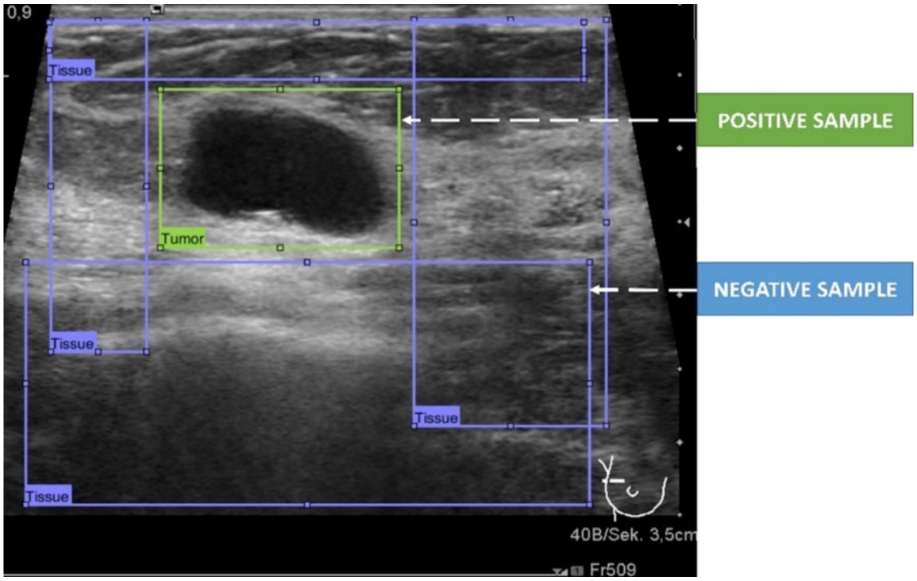

**Figure S8.** Recall-IoU and recall-LE characteristics resulting from the evaluation of the best breast lesion detection algorithm on the validation set. (A-i) YOLOv3 detection functions trained with transfer leraining by ‘fine tuning’ scored with different IoU thresholds; (A-ii) YOLOv3 detection functions trained with transfer leraining by ‘fine tuning’ scored with different LE thresholds; (B-i) YOLOv3 detection functions trained with transfer leraining by ‘fine tuning’ scored with different IoU thresholds; (B-ii) YOLOv3 detection functions trained with transfer leraining by ‘fine tuning’ scored with different LE thresholds; (C-i) Viola-Jones detection functions scored with different IoU thresholds; (C-ii) Viola-Jones detection functions scored with different LE thresholds.

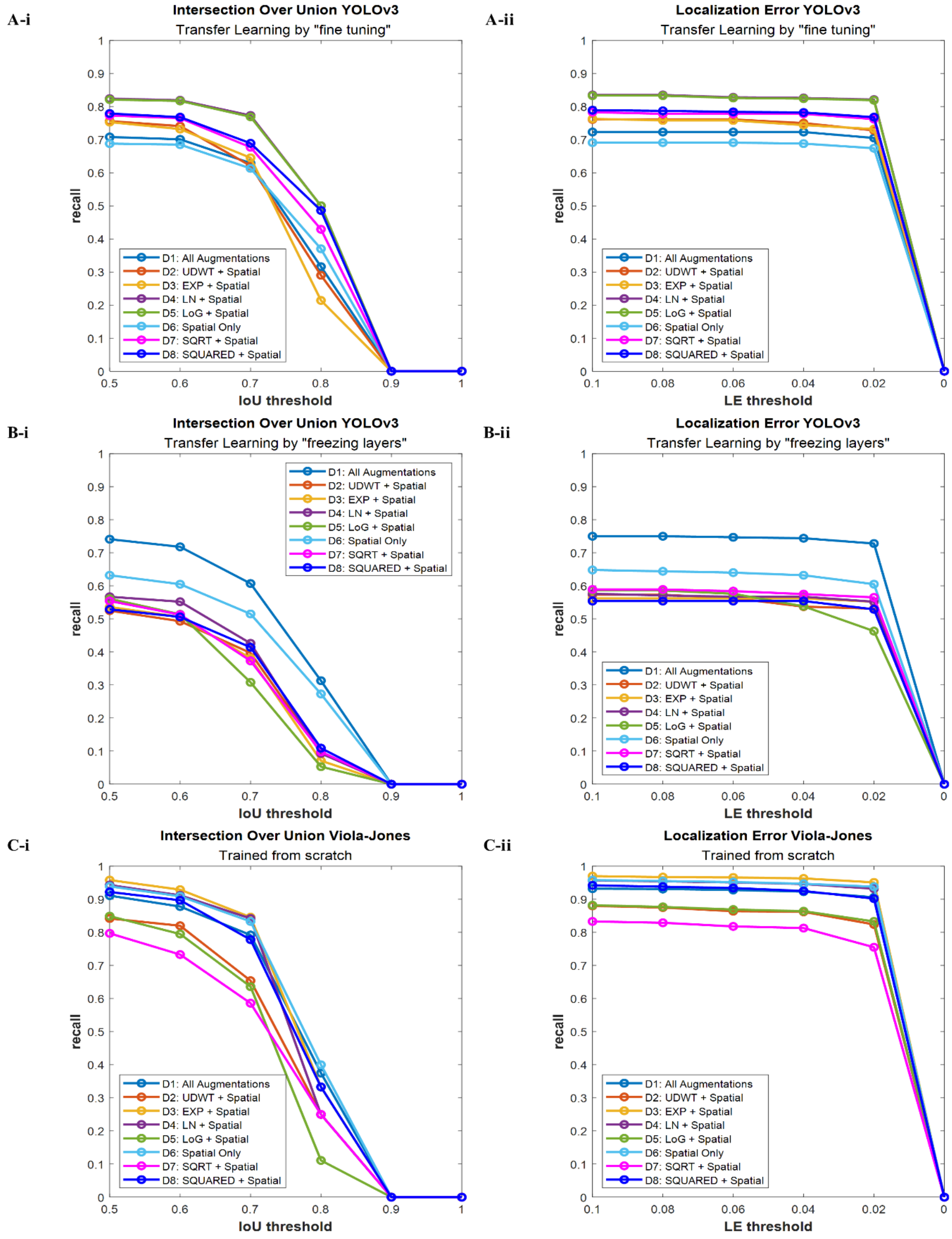

**Figure S9.** Recall-IoU and recall-LE characteristics resulting from the evaluation of the best breast lesion detection algorithm on the second data pool. (A-i) YOLOv3 detection functions scored with different IoU thresholds; (A-ii) YOLOv3 detection functions scored with different LE thresholds; (B-i) Viola-Jones detection functions scored with different IoU thresholds; (B-ii) Viola-Jones detection functions scored with different LE thresholds.

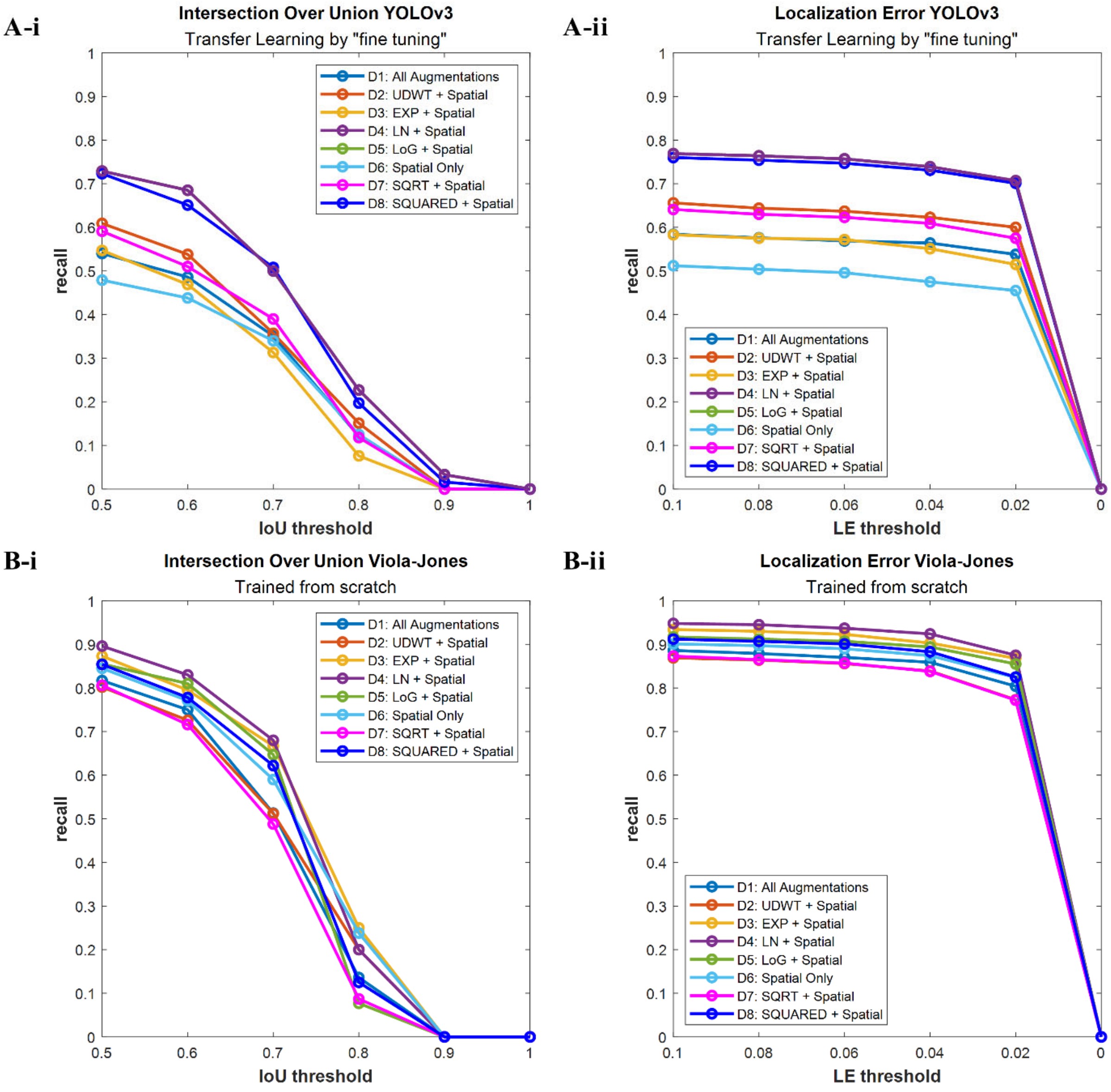

Supplement: Supplementary file 1 [file cancers-14-00277-s001.zip › cancers-1539454-supplementary.pdf]
